# Differentiating distinct and converging neural correlates of types of systemic environmental exposures

**DOI:** 10.1002/hbm.25783

**Published:** 2022-01-22

**Authors:** Teresa G. Vargas, Katherine S. F. Damme, Vijay A. Mittal

**Affiliations:** ^1^ Department of Psychology Northwestern University Evanston Illinois USA; ^2^ Department of Psychiatry Northwestern University Evanston Illinois USA; ^3^ Department of Medical Social Sciences Northwestern University Evanston Illinois USA; ^4^ Institute for Innovations in Developmental Sciences Northwestern University Evanston Illinois USA; ^5^ Institute for Policy Research Northwestern University Evanston Illinois USA

**Keywords:** chronic stress, development, environment, neural, systemic factors

## Abstract

Systemic environmental disadvantage relates to a host of health and functional outcomes. Specific structural factors have seldom been linked to neural structure, however, clouding understanding of putative mechanisms. Examining relations during childhood/preadolescence, a dynamic period of neurodevelopment, could aid bridge this gap. A total of 10,213 youth were recruited from the Adolescent Brain and Cognitive Development study. Self‐report and objective measures (Census and Federal bureau of investigation metrics extracted using geocoding) of environmental exposures were used, including *stimulation* indexing lack of safety and high attentional demands, *discrepancy* indexing social exclusion/lack of belonging, and *deprivation* indexing lack of environmental enrichment. Environmental measures were related to cortical thickness, surface area, and subcortical volume regions, controlling for other environmental exposures and accounting for other brain regions. Self‐report (|β| = .04–.09) and objective (|β| = .02–.06) environmental domains related to area/thickness in overlapping (e.g., insula, caudal anterior cingulate), and unique regions (e.g., for *discrepancy*, rostral anterior and isthmus cingulate, implicated in socioemotional functions; for *stimulation*, precuneus, critical for cue reactivity and integration of environmental cues; and for *deprivation*, superior frontal, integral to executive functioning). For *stimulation* and *discrepancy* exposures, self‐report and objective measures showed similarities in correlate regions, while *deprivation* exposures evidenced distinct correlates for self‐report and objective measures. Results represent a necessary step toward broader work aimed at establishing mechanisms and correlates of structural disadvantage, highlighting the relevance of going beyond aggregate models by considering types of environmental factors, and the need to incorporate both subjective and objective measurements in these efforts.

## Graphical abstract

Domains of systemic inequities and environmental factors were explored and related to gray matter morphometry in a nationally representative sample of youth. Self‐report (|β |= .04–.09) and objective (|β| = .02–.06) environmental domains related to area/thickness in overlapping (e.g., insula, caudal anterior cingulate), and unique regions (e.g., for discrepancy, rostral anterior and isthmus cingulate, implicated in socioemotional functions; for stimulation, precuneus, critical for cue reactivity and integration of environmental cues; and for deprivation, superior frontal, integral to executive functioning). Results represent a necessary step toward broader work aimed at establishing mechanisms and correlates of structural disadvantage, highlighting the relevance of going beyond aggregate models by considering types of environmental factors, and the need to incorporate both subjective and objective measurements in these efforts.

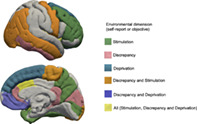

## 1 INTRODUCTION

Beyond the individual, the larger environmental and social context (i.e., systemic‐ and structural‐level environmental factors, including local, neighborhood, regional, or even country‐level characteristics) has been shown to impact physical and mental health, among other critical lifestyle outcomes (Arcaya et al., 2016; Laraia et al., 2012; Ludwig et al., 2012). Yet, most investigations have focused on adult populations (Arcaya et al., 2016). Much less is known about the effect of exposures during the dynamic developmental period of late childhood and preadolescence (Leventhal & Dupéré, 2019). Further, relative to individual‐level exposures (such as childhood trauma, life events, and bullying exposure), structural or systems level environmental factors have received relatively less attention in the literature—this is especially the case with regards to identifying putative biological or developmental mechanisms related to these factors. The existing literature suggests different dimensions of environmental exposures could relate to both convergent and distinct neural structures across neurodevelopment (McLaughlin, Sheridan, Humphreys, Belsky, & Ellis, 2021; Vargas, Conley, & Mittal, 2020). Neural correlates, while theorized, have yet to be tested and thus remain poorly understood. Further, understanding neural correlates of different structural exposures is ultimately crucial from an epidemiological and etiological standpoint (Minh, Muhajarine, Janus, Brownell, & Guhn, 2017). Improving existing conceptualizations of systemic barriers to healthy development stands to inform health policy, as well as prevention and intervention efforts at the societal level.

Chronic stress has long been identified as a central vulnerability factor toward a host of mental, physical health, and life outcomes (Bauer, 2008; Juster, McEwen, & Lupien, 2010; Lupien, Juster, Raymond, & Marin, 2018; McEwen, 2017). Classically, individual‐level stressors including childhood trauma, bullying, and parental conflict have been extensively studied with regards to underlying neural and biological mechanisms (Ellis, Boyce, Belsky, Bakermans‐Kranenburg, & Van IJzendoorn, 2011; McLaughlin, Sheridan, & Lambert, 2014). The literature on individual stressors and neural correlates has allowed for a more nuanced understanding of mechanisms of influence, along with pinpointing possible intervention and prevention targets. In contrast to the established research on individual stressors, the broader environmental and social context (i.e., systemic or structural level factors) has been relatively understudied with regards to neural correlates (Bronfenbrenner & Morris, 1998; Glass & McAtee, 2006).

Characteristics of the broader environment, particularly factors such as neighborhood poverty, exposure to crime, population density, and crime exposure, could be disadvantageous at a systemic level and have downstream impacts on the individual. Indeed, neighborhood and structural characteristics have been reliably associated with adverse health outcomes and alterations in physical development (Arcaya et al., 2016; Leventhal & Dupéré, 2019; Ludwig et al., 2012). Identifying systems level characteristics could aid efforts to understand systemic inequities and disadvantage faced by marginalized groups in the United States. However, efforts to identify neural and biological factors that relate to these systemic exposures have been sparse. Enriching the existing literature with a neural account of different systemic environmental exposures would result in a more integrative perspective on environmental factors. In fact, a neural model of systemic exposures may aid in identifying putative mechanisms underlying the impact of different exposures.

Exposure to individual or systemic environmental factors can have an independent impact through both stress exposure and alterations in neural development and as a result do not require conscious awareness of exposure having occurred to be impactful (McLaughlin et al., 2021). As a result, studying neural correlates of systemic environmental factors during critical developmental periods provides a crucial perspective. Childhood and preadolescence constitute a dynamic period for neural development, particularly for gray matter structure (Jeon, Mishra, Ouyang, Chen, & Huang, 2015; Lyall et al., 2015; Tamnes et al., 2017; Wierenga, Langen, Oranje, & Durston, 2014). During late childhood, gray matter features are undergoing foundational developmental processes (Jeon et al., 2015; Wierenga et al., 2014). Widespread gray matter volume decreases are taking place; in addition, the developmental timing of volume, thickness, and surface area varies by cortical region, and cortical thickness and surface area develop independently of one another (Wierenga et al., 2014). Along with total gray matter volume decreases, cortical thinning and pruning processes are particularly active during adolescence (Norbom et al., 2021).

The marked neural reorganization occurring at this age yields greater plasticity, or sensitivity to environmental influences (Nelson III & Gabard‐Durnam, 2020; Pechtel & Pizzagalli, 2011). As such, these gray matter metrics could provide unique insights into developmental processes and underlying biological mechanisms that are influenced by environmental factors. Cortical thickness may index synaptic pruning, cell shrinkage, apoptosis, and dendritic arborization (Jeon et al., 2015; Tamnes et al., 2017). Surface area could reflect processes related to cortical folding and gyrification (Garcia, Kroenke, & Bayly, 2018). Collectively these metrics could provide unique insights for emerging types of environmental vulnerability in the years prior to adolescence and young adulthood, when other contextual, interpersonal, and neurodevelopmental vulnerabilities could compound pre‐existing risk factors. Different types of exposures could require different considerations with regards to prevention and intervention efforts for mental and physical health vulnerability. As such, understanding neural correlates of types of environmental exposures is a crucial first step.

As the largest study to date on adolescent development, the adolescent brain and cognitive development (ABCD) study**®** provides an excellent opportunity to further understand these questions (Casey et al., 2018). Indeed, existing literature has already yielded insights into the subject, relating environmental factors to neural structure and function. Existing studies have found relations between broader neighborhood disadvantage, resting state and structural features (Hackman et al., 2021; Rakesh, Seguin, Zalesky, Cropley, & Whittle, 2021). Other work harnessing ABCD has focused on neural correlates of single features, that is, neighborhood deprivation (Mullins, Campbell, & Hogeveen, 2020; Taylor, Cooper, Jackson, & Barch, 2020; Vargas, Damme, & Mittal, 2020). Studies distinguishing types of systemic environmental exposures are sparser. To our knowledge, one study explored distinct types of environmental exposures (using neighborhood features acquired through geocoding participant addresses), along with relations to brain structure (Karcher, Schiffman, & Barch, 2021). However, this study examined aggregate brain metrics of cortical thickness, volume and surface area, curtailing the ability to make inferences with regards to specific neural correlates and mechanisms underlying each environmental exposure. Investigations seeking to establish specificity of systemic environmental exposure on neurodevelopment are thus needed to enrich current conceptualizations of environmental vulnerability and putative neural correlates, as a first step toward understanding possible mechanisms.

**FIGURE 1:**
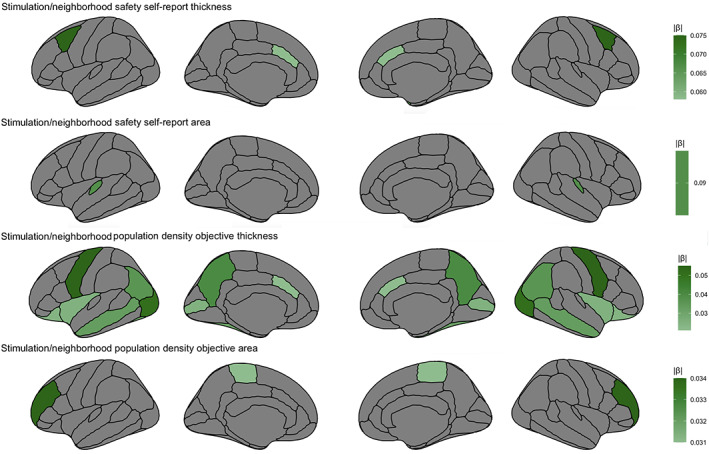
Relations between self‐report and objective measures for the stimulation domain, area, and thickness regions that are significant (*p* < .05) while controlling for other regions, accounting for other domains, age, sex, family, and scanner

To this end, a recent review delineated three systemic environmental exposure dimensions based on available evidence from epidemiological and neuroscience literature (Vargas, Conley, & Mittal, 2020). The resulting stimulation, deprivation, and discrepancy (SDD) model posed three environmental exposure dimensions that are theorized to confer both converging and distinguishable effects on neural structure (figure 1 in the study by Vargas et al., 2020; Vargas, Damme, Osborne, & Mittal, 2021). The environmental dimensions include *stimulation* exposures, with intermediary mechanisms of high sensory demands and lack of safety (e.g., high neighborhood crime and population density), *discrepancy* exposures, with intermediary mechanisms of social exclusion, low social capital and lack of belonging (e.g., high neighborhood income inequality), and *deprivation* exposures with intermediary mechanisms of lack of environmental enrichment (e.g., neighborhood median family income). In an earlier study, the specificity of the environmental domains in the SDD theory was tested through exploratory and confirmatory factor analyses. Environmental dimensions were distinguishable and related to vulnerability to psychopathology (Vargas et al., 2021). As such, some support has been found in the ABCD data for the distinctness of the domains.

Although the review outlined theorized neural regions that could be specific to each domain, these hypotheses have yet to be directly tested. As mentioned earlier, childhood and preadolescence are prime periods of marked environmental sensitivity, characterized by widespread neural plasticity and gray matter development. As such, efforts to understand these environmental factors and neural correlates during the childhood and preadolescence developmental period could contribute to crucial intervention and prevention efforts. These systemic exposures could only confer a generalized effect on neural structure, as found in the study by Karcher et al. (2021). Or, the environmental dimensions could relate specifically to certain regions, while also exhibiting broader effects at the whole brain level (as hypothesized by the SDD theory). Limited research testing these questions with different environmental exposures in the same sample hinder ability to further clarify these matters. As such, the current study marks the first explicit test of neural correlates of these three domains together, accounting for unique influences over and above other domains.

The present study sought to explore gray matter neural correlates for environmental dimensions of *stimulation*, *discrepancy*, and *deprivation*. First, self‐report and subjective measures were identified, consistent with the SDD model. Then, subsamples were created based on the top 25 percentile of exposure to the environmental factors. Finally, to establish specificity over and above general neighborhood disadvantage, cortical thickness and surface area, and subcortical volumes were used to predict exposure to the self‐report and objective environmental dimensions, while accounting for other cortical and subcortical regions, and for exposure to other domains. Controlling for all cortical/subcortical regions within each analysis allowed for exploring SDD theory specificity predictions of certain regions over and above other regions (see supplementary material). Taken together, the current analyses allow for an opportunity to understand theorized environmental dimensions and neural correlates.

## 2 MATERIALS AND METHODS

### 2.1 Self‐report questionnaires

Self‐report scales relevant to the three domains were chosen across administered scales (Vargas et al., 2020). Self‐report measures were developed by the ABCD team to index environmental and cultural factors that could be relevant to development (Alegria et al., 2004; Zucker et al., 2018). As such, these measures index structural factors/exposures that occur at the systems level (Table 1) (Vargas et al., 2020).

**TABLE 1:** Self‐report scales used for subjective measures of environmental exposures, along with domains each measure represents

Scale name	Citation	Domain
ABCD Parent Multi‐Group Ethnic Identity‐Revised Survey (MEIM)	Phinney and Ong (2007)	Discrepancy
ABCD Parent Vancouver Index of Acculturation (VIA)—Short Survey	Ryder, Alden, and Paulhus (2000)	Discrepancy
ABCD Parent neighborhood safety/crime survey modified from PhenX (NSC)	Echeverria, Diez‐Roux, and Link (2004) and Mujahid, Diez Roux, Morenoff, and Raghunathan (2007)	Stimulation
ABCD Parents Demographics survey	Garavan et al. (2018)	Deprivation

### 2.2 Objective neighborhood features

Residential history was collected through addresses where participants had lived across their lifetime. Addresses were used to determine census tracts corresponding to each location. Each tract represents census‐delineated neighborhoods. Census and Federal Bureau of Investigation (FBI) data were used to calculate neighborhood population density, total crimes occurring in certain neighborhood, average neighborhood income inequality (i.e., higher neighborhood income inequality meaning that higher income individuals receive much larger percentages of the total income in a given neighborhood), and median family income. Since these metrics are compiled based on government data, they will be referred to as “objective neighborhood features,” drawing a contrast from neighborhood features of interest that are also assessed through self‐report, such as the ABCD Parent neighborhood safety/crime survey (NSC). See Table 2 for further description of values for objective and subjective measures.

**TABLE 2:** Demographic characteristics

					Age M (*SD*)	Sex (% male)	Race/ethnicity^a^
Self‐report factor sample	Stimulation/neighborhood safety M (*SD*)^b^	Discrepancy/sense of belonging M (*SD*)^c^	Discrepancy/American culture participation M (*SD*)^d^	Deprivation/deprivation M (*SD*)^e^			
Stimulation/neighborhood safety (*n* = 2,105)	7.891 (2.092)	21.288 (5.225)	52.067 (13.381)	0.796 (1.336)	9.903 (0.625)	51.211	27.886
Discrepancy/sense of belonging (*n* = 2,275)	11.684 (2.874)	15.389 (3.156)	51.929 (13.354)	0.412 (1.005)	9.902 (0.623)	51.648	57.407
Discrepancy/American culture participation (*n* = 1,856)	10.769 (3.148)	19.688 (5.324)	38.732 (8.800)	0.668 (1.253)	9.884 (0.613)	52.047	37.446
Deprivation/deprivation (*n* = 1,493)	10.102 (3.254)	21.528 (5.406)	51.944 (13.675)	2.096 (1.271)	9.883 (0.628)	54.186	24.180

Objective factor sample	Stimulation/crimes^f^	Stimulation/population density^g^	Discrepancy/income inequality^h^	Deprivation/median family income^i^			
Stimulation/crimes (*n* = 2,249)	167,979.554 (121,609.968)	4,081.275 (4,497.255)	2.438 (1.240)	70,422.004 (36,703.363)	9.922 (0.611)	50.778	35.216
Stimulation/population density (*n* = 2,553)	109,684.446 (125,617.048)	5,021.263 (3,931.634)	2.793 (1.264)	61,776.675 (34,582.331)	9.908 (0.627)	52.605	30.121
Discrepancy/income inequality (*n* = 2,566)	61,607.632 (91,319.251)	3,303.578 (3,297.845)	3.886 (0.807)	41,136.815 (17,229.065)	9.886 (0.616)	50.624	25.253
Deprivation/median family income (*n* = 2,554)	69,738.807 (101,840.166)	3,435.006 (3,018.204)	3.700 (1.004)	36,649.972 (9,434.837)	9.884 (0.620)	51.684	20.360

^a^ % white, non‐latine/o/a.

^b^ Higher values indicate higher feelings of safety within one's neighborhood (3 items ranging from 1 to 5, with 1 meaning strongly disagree and 5 meaning strongly agree), scores range from 3 to 15.

^c^ Higher values indicate lower sense of belonging with ethnic group (6 items ranging from 1 to 5, with 1 meaning strongly disagree and 5 meaning strongly agree), scores range from 6 to 30.

^d^ Higher values indicate higher participation in American culture (8 items ranging from 1 to 9, with 9 meaning completely agree), scores range from 8 to 72.

^e^ Higher scores indicate greater degrees of deprivation (7 items ranging from 0 to 1, with 0 meaning no and 1 meaning yes), scores range from 0 to 7.

^f^ Values indicate number of total crimes recorded within the Census‐delineated neighborhood.

^g^ Values indicate number of people per square mile.

^h^ Values for income inequality represent the log of 100 × ratio of the number of households with <10,000 annual income to the number of households with >50,000 annual income (Singh, 2003), higher values represent higher income inequality at the neighborhood level. Values range from −1.13 to 8.16.

^i^ The median of the yearly household income in U.S. dollars for households in a given neighborhood.

### 2.3 Theorized systemic environmental exposure domains

Chosen variables indexed exposures to environmental factors occurring at the systems level. For the *stimulation* domain, high crime regions, along with urban/areas with high population density, have been theorized to comprise high attentional demands, engaging threat neural correlates and conferring higher arousal of stress systems (Freeman et al., 2015; Gong, Palmer, Gallacher, Marsden, & Fone, 2016; Newbury et al., 2017). As such, the NSC survey was chosen, which assesses neighborhood safety. For objective measures, neighborhood total crimes and population density were chosen as part of the *stimulation* domain. For *discrepancy*, the ABCD Parent Multi‐Group Ethnic Identity‐Revised Survey (MEIM) and ABCD Parent Vancouver Index of Acculturation (VIA) scales were used, consistent with evidence that a lack of sense of belonging within one's culture, along with lack of participation and engagement with the majority culture and with one's culture, are cultural/systems level factors that can confer a lack of social capital and social exclusion (Emerson, Minh, & Guhn, 2018; Veling et al., 2008; Yang, Lei, & Kurtulus, 2018). For objective measures within the *discrepancy* domain, neighborhood income inequality was chosen, given evidence of high income inequality being linked to lack of belonging and feeling of social exclusion, consistent with the *discrepancy* domain (Vargas et al., 2021; Vargas et al., 2020). For the *deprivation* domain, the ABCD parent's demographic survey was used to index lack of access to environmental enrichment (with questions probing for access to resources such as access to doctors if needed, food, and utilities; Table S1). For objective measures, neighborhood median family income was used as a measure of neighborhood deprivation.

### 2.4 Structural MRI

Participants completed a high‐resolution T1‐weighted structural MRI scan (1‐mm isotropic voxels) using scanners from GE Healthcare (Waukesha, Wisconsin), Philips Healthcare (Andover, Massachusetts), or Siemens Healthcare (Erlangen, Germany) (Casey et al., 2018). Structural MRI data were processed using FreeSurfer version 5.3.0 (http://surfer.nmr.mgh.harvard.edu/) (Fischl, Sereno, Tootell, & Dale, 1999) according to the standard processing pipelines (Casey et al., 2018). Processing included removal of nonbrain tissue, segmentation of gray and white matter structures (Fischl et al., 2002), and cortical parcellation. All scan sessions underwent radiological review whereby scans with incidental findings were identified and excluded. Quality control for the structural images comprised visual inspection of T1 images and Free‐Surfer outputs for quality (Hagler et al., 2019). Subjects whose scans failed inspection (due to severe artifacts or irregularities) were excluded (Figure S1). The Desikan‐Killiany Atlas was used for cortical parcellation (Hagler et al., 2019). For subcortical parcellation, the Aseg atlas was used (Fischl et al., 2002).

### 2.5 Sample selection

The ABCD study was designed to recruit a nationally representative sample of youth. See Garavan et al. (2018) for more information on demographics and Karcher and Barch (2021) for information on psychopathology measures. For subjective environmental factors, the sample from our group's earlier work delineating factors within the SDD theory was used, including 7,443 participants with available subjective environmental domain data (Vargas et al., 2021). The initial sample for objective environmental factors included 10,213 participants who had available structural imaging and geocoded data. To rule out participants with a complete lack of exposure to environmental risk factors (e.g., participants that endorsed zero exposure to deprivation), subsamples were created for each domain for participants with scores in the top 25 percentile for environmental risk (associated with symptoms in earlier work, i.e., high deprivation, high crime and population density, low sense of neighborhood safety, low sense of belonging, and low American/majority culture participation; Figure S2). The 25th percentile was used given the SDD theory's emphasis on understanding systemic vulnerability factors. As most of the sample did not endorse significant exposure to these environmental dimensions, the study focused on those individuals that did, in order to assess relations between gray matter morphology and substantial exposure to systemic vulnerability factors. The literature on neighborhood factors has frequently adopted this approach in order to understand the impact of greater levels of exposure, which would likely be the targets of systems level intervention and prevention efforts (Crump, Sundquist, Sundquist, & Winkleby, 2011; Cummins, McKay, & MacIntyre, 2005; Lang et al., 2009; Major et al., 2010; Pearce, Witten, Hiscock, & Blakely, 2007; Vos, Posthumus, Bonsel, Steegers, & Denktaş, 2014). As such, the study remained consistent to this literature by focusing on folks with greater levels of exposure. The 25th percentile cutoffs for self‐report factors were as follows: 10 for stimulation/neighborhood safety (*n* = 2,105), 18 for discrepancy/sense of belonging (*n* = 2,275), 48 for discrepancy/American culture participation (*n* = 1,856), and 1 for deprivation/deprivation (*n* = 1,493). The 25th percentile cutoffs for objective factors were as follows: 53,400 for stimulation/neighborhood crimes (*n* = 2,249), 2,814.3 for stimulation/population density (*n* = 2,553), 2.95 for discrepancy/income inequality (*n* = 2,566), and 50,357 for deprivation/median family income (*n* = 2,554; Table 2, Tables S2–S10). Analyses on self‐report features from our prior work accounted for exposures to other self‐report domains. Similarly, analyses on objective features accounted for exposures to other objective domains. Analyses were run on the entire sample as well, and while not the focus of the study, these are presented in the supplementary material (Tables S11–S19).

### 2.6 Cortical area/thickness and subcortical volumes as predictors of self‐report and objective environmental domain exposures

Mixed effect models were run using the nlme version 3.1.148 (Pinheiro et al., 2021) package in r version 4.0.2. Desikan‐Killiani Atlas bilateral (averaged across hemispheres to avoid collinearity concerns due to high correlations among left and right sides of the same region) cortical regions (34 total) were used as predictors in a single varying‐intercepts mixed effect model accounting for age, sex and other self‐report/objective domains as fixed effects, and family and scanner as random effects, with self‐report and objective environmental exposures as outcome variables. Environmental domains were treated as independent, and all regions and components were in the same model. Analyses were run for cortical thickness and surface area separately, given their earlier mentioned distinct developmental trajectories and underlying neural structural indicators. Analyses were run such that one model included all 34 of the Desikan‐Killiany‐delineated bilateral regions as predictors. As such, results presented accounted for and corrected for all other brain regions within the same model. Prior to analyses, variables were converted to standardized units (*z* scores). Standardized brain metrics/predictors did not correlate highly with each other (*r*s were below .5, in a vast majority of cases under .1); as such, collinearity due to brain metric predictors was not a concern in models that were run. The car package, version 3.0.11 was used to calculate variance inflation factor to further assess for multicollinearity; values were below 5, meeting conventional thresholds (Fox, 2015).

### 2.7 Subcortical volumes as predictors of self‐report and objective environmental domain exposures

As described above, aseg Atlas subcortical regions (seven total) were used as predictors in a single varying‐intercepts mixed effect model accounting for age, sex and other self‐report/objective domains as fixed effects, and family and site as random effects, with stimulation/discrepancy/deprivation subjective and objective measures as outcome variables.

### 2.8 Data analytic strategy

Prior to analyses, variables were converted to standardized units (*z* scores). Results were visualized using r packages ggseg version 1.6.3, and ggseg3d version 1.6.3 (Mowinckel & Vidal‐Piñeiro, 2020). See supplementary material for coefficient values, standard errors, and *p* values for model predictors. A series of distinct theories/models were tested in the present study to determine whether self‐report and objective systemic/environmental dimensions (stimulation, discrepancy, and deprivation), would relate to gray matter morphology (Armstrong, 2014; Cabin & Mitchell, 2000; Fiedler, Kutzner, & Krueger, 2012): there were three distinct theoretically grounded tests (for stimulation, discrepancy, and deprivation), which were separately predicted for self‐report and objective measures. The measures used were grounded in previous research that identified domains using factor analyses and related them to vulnerability for psychopathology (Vargas et al., 2021). In addition to correcting for all cortical and subcortical regions within the same model, further correction for comparisons was conducted for each gray matter metric tested (cortical thickness, surface area, and subcortical volume) using Bonferroni thresholds for three tests of one similar hypothesis, resulting in a threshold of 0.016 (see Table 3) (Bonferroni, 1936; Shaffer, 1995).

**TABLE 3:** Summary of associations that passed Bonferroni correction

Stimulation domain				
Region	Metric	Measure	*p* value	Bonferroni correction^a^
Caudal anterior cingulate	Thickness	Neighborhood safety	.019	
Temporal pole	Thickness	Neighborhood safety	.010	✓
Caudal middle frontal	Thickness	Neighborhood safety	.044	
Transverse temporal	Area	Neighborhood safety	.007	✓
Accumbens	Volume	Neighborhood safety	.020	
Caudal anterior cingulate	Thickness	Neighborhood population density	.016	✓
Lateral orbitofrontal	Thickness	Neighborhood population density	.020	
Par striangularis	Thickness	Neighborhood population density	.018	
Precentral	Thickness	Neighborhood population density	.0002	✓
Paracentral	Area	Neighborhood population density	.010	✓
Rostral middle frontal	Area	Neighborhood population density	.027	
Inferior parietal	Thickness	Neighborhood population density	.033	
Precuneus	Thickness	Neighborhood population density	.003	✓
Fusiform	Thickness	Neighborhood population density	.021	
Middle temporal	Thickness	Neighborhood population density	.033	
Lateral occipital	Thickness	Neighborhood population density	.002	✓
Pericalcarine	Thickness	Neighborhood population density	.021	
Insula	Thickness	Neighborhood population density	.013	✓

Discrepancy domain				
Insula	Thickness	Sense of belonging	.002	✓
Cuneus	Area	Sense of belonging	.036	
Insula	Thickness	American culture participation	.032	
Inferior parietal	Area	American culture participation	.015	✓
Precentral	Thickness	American culture participation	.002	✓
Isthmus cingulate	Area	American culture participation	.003	✓
Paracentral	Thickness	American culture participation	.010	✓
Middle temporal	Area	Neighborhood income inequality	.043	
Inferior temporal	Thickness	Neighborhood income inequality	.043	
Lateral occipital	Thickness	Neighborhood income inequality	.040	
Caudal anterior cingulate	Area	Neighborhood income inequality	.025	
Medial orbitofrontal	Area	Neighborhood income inequality	.002	✓
Rostral anterior cingulate	Area	Neighborhood income inequality	.008	✓
Caudate	Volume	Neighborhood income inequality	.037	
Hippocampus	Volume	Neighborhood income inequality	.027	

Deprivation domain				
Caudal anterior cingulate	Area	Deprivation	.015	✓
Medial orbitofrontal	Thickness	Neighborhood median family income	.038	
Superior frontal	Thickness	Neighborhood median family income	.005	✓
Insula	Area	Neighborhood median family income	.045	

^a^ Threshold set at 0.0167, ✓ indicates the association passed Bonferroni correction.

## 3 RESULTS

### 3.1 Stimulation self‐report factors and objective measures

Lower caudal anterior cingulate (β = −.058, *t* = −2.38, *p* = .019, 95% CI −0.106 to −0.010; Figure 1) and temporal pole (β = −.067, *t* = −2.62, *p* = .010, 95% CI −0.117 to −0.016) thickness related to greater neighborhood safety. Greater caudal middle frontal thickness (β = −.058, *t* = 2.04, *p* = .044, 95% CI 0.002–0.147), on the other hand, related to greater neighborhood safety. For area, lower transverse temporal (β = −.086, *t* = −2.772, *p* = .007, 95% CI −0.148 to −0.025) area related to increased neighborhood safety. Subcortically, higher neighborhood safety related to lower accumbens volume (β = −.065, *t* = −2.351, *p* = .020, 95% CI −0.120 to −0.10).

The stimulation/neighborhood total crimes objective measure did not relate to specific cortical or subcortical regions over and above other cortical/subcortical regions. Within frontal lobe, greater caudal anterior cingulate (β = .021, *t* = 2.421, *p* = .016, 95% CI 0.004–0.039), lateral orbitofrontal (unique to *stimulation* domain; β = .024, *t* = 3.125, *p* = .020, 95% CI 0.003–0.045), and par striangularis (also unique to *stimulation*; β = .029, *t* = 2.384, *p* = .018, 95% CI 0.005–0.053) thickness predicted greater population density. Lower precentral thickness (β = −.055, *t* = −3.850, *p* = .0002, 95% CI −0.083 to −0.027) also predicted greater population density. With regards to area, lower paracentral (β = −.031, *t* = −2.614, *p* = .010, 95% CI −0.055 to −0.008) and rostral middle frontal (β = −.034, *t* = −2.229, *p* = .027, 95% CI −0.063 to −0.004) area related to greater population density.

Within parietal lobe, lower inferior parietal (β = −.036, *t* = −2.150, *p* = .033, 95% CI −0.070to 0.003) and greater precuneus (β = −.040, *t* = 3.022, *p* = .003, 95% CI 0.01–0.066) thickness predicted greater population density. For temporal regions, greater fusiform (β = .031, *t* = 2.333, *p* = .021, 95% CI 0.005–0.057) and middle temporal (β = .033, *t* = 2.148, *p* = .033, 95% CI 0.003–0.064) thickness predicted greater population density. For occipital lobe, greater lateral occipital (β = .051, *t* = 3.125, *p* = .002, 95% CI 0.019–0.083) and lower pericalcarine (β = −.025, *t* = −2.321, *p* = .021, 95% CI −0.046 to −0.004) thickness predicted higher population density. Finally, within limbic regions, lower insula (β = −.025, *t* = −2.502, *p* = .013, 95% CI −0.045 to −0.005) thickness predicted higher population density.

### 3.2 Discrepancy self‐report factors and objective measures

**FIGURE 2:**
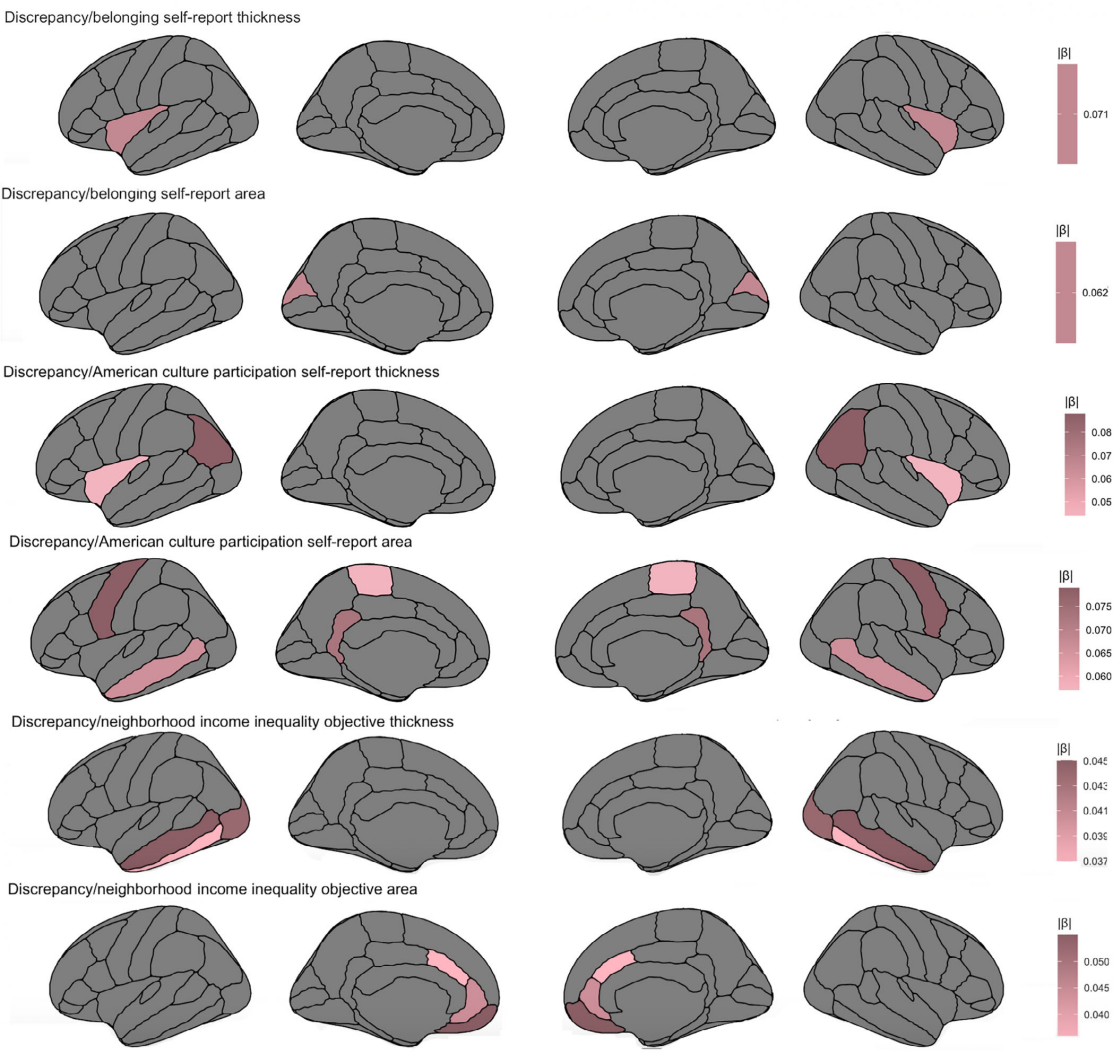
Relations between self‐report and objective measures for the discrepancy domain, area, and thickness regions that are significant (*p* < .05) while controlling for other regions, accounting for other domains, age, sex, family, and scanner

Greater insula thickness (β = −.071, *t* = −3.143, *p* = .002, 95% CI −0.116 to −0.027; Figure 2) and lower cuneus area (β = .062, *t* = 2.108, *p* = .036) related to a lower sense of belonging with ethnic group. Lower insula (β = .044, *t* = 2.158, *p* = .032, 95% CI 0.004–0.085) and inferior parietal (β = .088, *t* = 2.470, *p* = .015, 95% CI 0.018–0.159) thickness related to lower American culture participation. Greater precentral area (β = .079, *t* = 3.132, *p* = .002, 95% CI 0.029–0.129) related to greater American culture participation. Conversely, lower isthmus cingulate (β = −.073, *t* = −2.991, *p* = .003, 95% CI −0.122 to −0.025) and paracentral (β = −.057, *t* = −2.595, *p* = .010, 95% CI −0.100 to −0.014) area related to greater American culture participation.

Higher middle temporal thickness related to greater income inequality (β = .045, *t* = 2.036, *p* = .043, 95% CI 0.001–0.090). Conversely, lower inferior temporal (β = −.037, *t* = −2.03, *p* = .043, 95% CI −0.073 to 0.001) and lateral occipital (β = −.044, *t* = −2.070, *p* = .040, 95% CI −0.085 to −0.002) thickness related to greater income inequality. For surface area, lower caudal anterior cingulate area related to increased income inequality (β = −.036, *t* = −2.254, *p* = .025, 95% CI −0.067 to 0.004). Greater medial orbitofrontal (β = .055, *t* = 3.103, *p* = .002, 95% CI 0.020–0.090) and rostral anterior cingulate (β = .044, *t* = 2.66, *p* = .008, 95% CI 0.011–0.076) area related to increased income inequality. Subcortically, lower caudate (β = −.030, *t* = −2.098, *p* = .037, 95% CI −0.059 to −0.002) and hippocampal (β = −.039, *t* = −2.230, *p* = .027, 95% CI −0.074 to −0.005) volume related to increased income inequality.

### 3.3 Deprivation self‐report factor and objective measures

**FIGURE 3:**
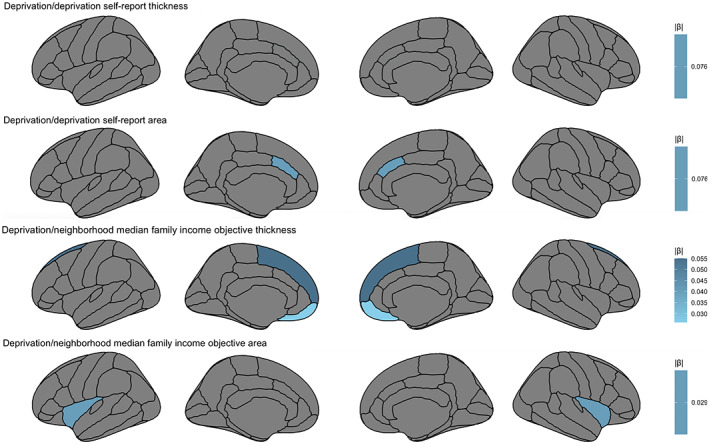
Relations between self‐report and objective measures for the deprivation domain, area, and thickness regions that are significant (*p* < .05) while controlling for other regions, accounting for other domains, age, sex, family, and scanner

Lower caudal anterior cingulate area related to higher levels of self‐reported *deprivation* (β = −.076, *t* = −2.457, *p* = .015, 95% CI −0.137 to −0.015; Figure 3). With regards to objective *deprivation* measures, higher medial orbitofrontal thickness (β = −.026, *t* = −2.091, *p* = .038, 95% CI −0.051 to −0.002) and insula area (β = −.029, *t* = −2.014, 95% CI −0.058 to −0.001, *p* = .045) related to lower neighborhood median family income. Unique to analyses in the *deprivation* domain, greater superior frontal (β = −.055, *t* = −2.822, *p* = .005, 95% CI −0.094 to −0.017) thickness related to lower neighborhood median family income.

## 4 DISCUSSION

**FIGURE 4:**
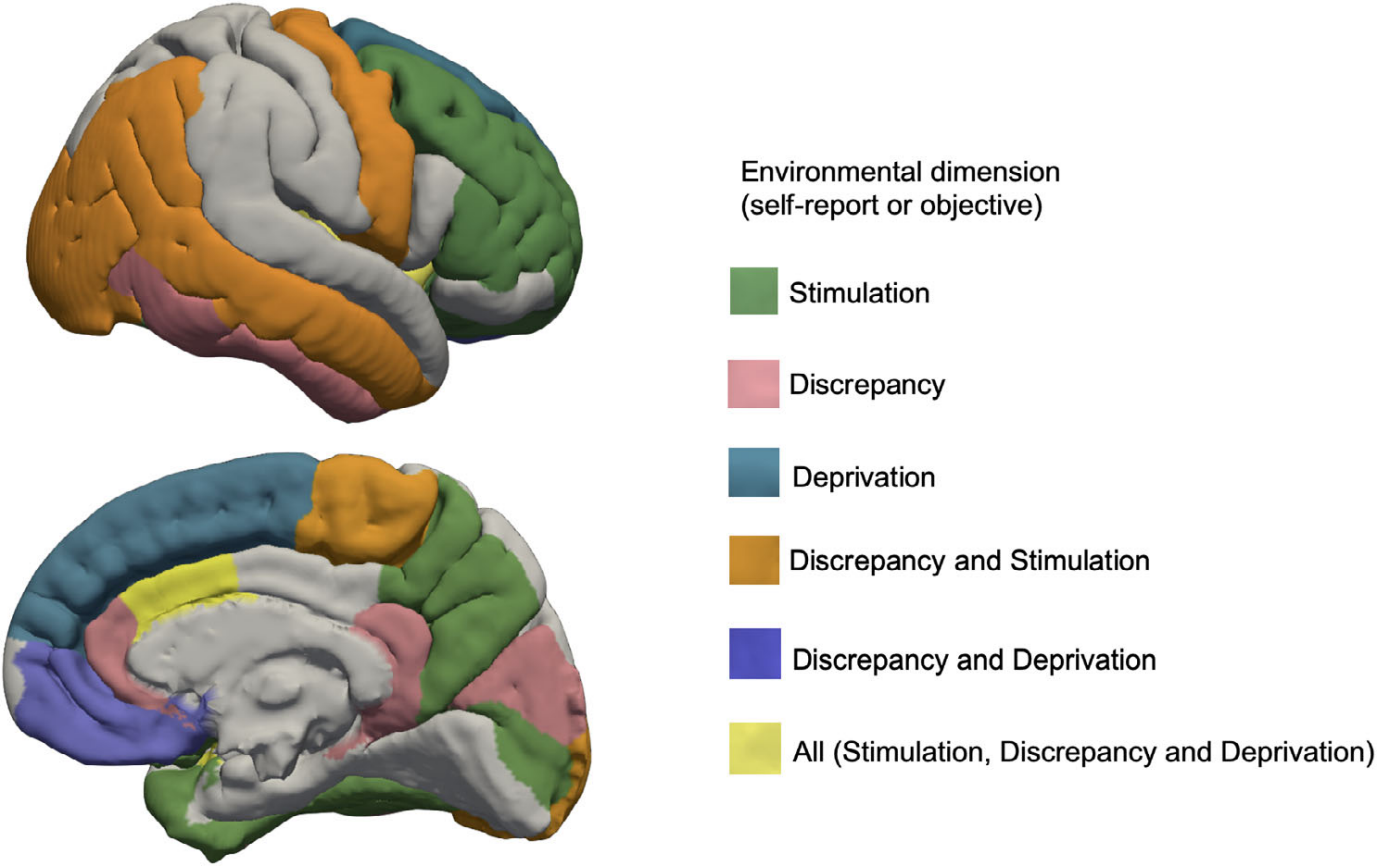
Summary of regions for cortical thickness and surface area that related to environmental dimensions individually, and regions that related to more than one domain independently. Highlighted regions were significant (*p* < .05) after controlling for other regions, age, sex, family, scanner, and exposure to other environmental domains

The current study explored dimensions of environmental exposures with relation to neural structure. Self‐report and objective measures were harnessed to test whether different environmental dimensions would relate to unique and converging gray matter features. *Stimulation* exposures related to prefrontal, temporal and parietal regions, which are implicated in threat processing, social cognitive processes, and sensory integration. *Discrepancy* exposures, on the other hand, related to cingulate, medial orbitofrontal and temporal regions, regions implicated in socioemotional processes related to social exclusion. Lastly, *deprivation* exposures related to thickness frontal regions, consistent with pruning hypotheses of deprivation, postulating that lack of exposure to rich environments could result in over‐pruning, thus having aggregating effects on neurodevelopment over time (Laraia et al., 2012; McLaughlin et al., 2014). Results were consistent across measures for the *stimulation* and *discrepancy* domains; caudal anterior cingulate thickness related to self‐report neighborhood safety and objective neighborhood population density, while insula thickness related to self‐report sense of belonging and self‐report American culture participation, and cingulate regions related to self‐report American culture participation and neighborhood income inequality. Notably, caudal anterior cingulate and insular regions uniquely predicted exposures across all three domains while accounting for other exposures, and for other brain regions. Taken together, results offer support for the notion that different types of systemic environmental factors and stressors may confer overlapping but also unique patterns of vulnerability on the developing brain (Figure 4).

### 4.1 Stimulation exposures

*Stimulation* exposures related to regions implicated in hypothesized intermediary mechanisms of lack of safety and high attentional demands. Precuneus thickness, critical for cue reactivity and integration of environmental cues, related to population density (Hebscher, Levine, & Gilboa, 2018). Within the dorsolateral prefrontal cortex (engaged during processing of threat related stimuli, shown to be impacted by threat exposure, and related to urban upbringing in adult samples), rostral middle frontal area predicted objective population density (Balderston, Hsiung, Ernst, & Grillon, 2017; Bishop, Duncan, Brett, & Lawrence, 2004; Haddad et al., 2015). Subcortically, accumbens volume, with relations to differentiating safety from threat cues, related to self‐report neighborhood safety, though rostral middle frontal and accumbens regions did not pass Bonferroni correction (Ray, Russ, Walker, & McDannald, 2020).

In addition to originally hypothesized regions, *stimulation* and *discrepancy* exposures were predicted by regions implicated in sensory processing, including auditory/language processing regions (transverse temporal), primary visual processing regions (lateral occipital), visual and semantic attention/integration (temporal pole), as well as some primary motor regions (precentral and paracentral). Consistent with stimulation, it is possible that high environmental attentional demands result in greater activity in regions involved in sensory processing and cue reactivity, altering neural structure during developmental sensitive periods (Ellis et al., 2011; Petanjek et al., 2011). Given that the current study is cross sectional, future investigations are needed to further test the impact of environmental complexity on sensory processing. It could be that some *discrepancy* exposures partially engage some intermediary mechanisms from *stimulation* exposures. For example, exposures related to low social capital, low sense of belonging, and social exclusion could engage threat circuitry and cue reactivity (Vargas et al., 2020). As such, results highlight the importance of conceptualizing environmental domains dimensionally, as well as of measuring kinds of exposures in the same sample. Future studies are needed to improve understanding of possible overlap of underlying neural mechanisms between domains. Investigations will also benefit from more targeted, granular measures for each exposure.

### 4.2 Discrepancy exposures

In tandem, *Discrepancy* exposures related to regions implicated in theorized intermediary mechanisms of social exclusion, lack of belonging and low social capital. Notably, isthmus cingulate, rostral and caudal anterior cingulate area related to self‐report American culture participation and objective neighborhood income inequality (with rostral anterior cingulate not surviving Bonferroni correction). Cingulate regions have been related to processing of social threat, rejection, and lack of belonging, along with pain related processing (Adolphs, 2009; Eisenberger & Cole, 2012; Eisenberger, Lieberman, & Williams, 2003). In addition, caudate regions, linked to social cognition and emotion processing, also related to neighborhood income inequality, though not surviving correction (Coan, Schaefer, & Davidson, 2006; Kemp et al., 2013; Weidt et al., 2016). Within the medial temporal lobe, medial prefrontal area, a region long implicated in affect regulation and social functions including understanding others' emotions, predicted objective neighborhood inequality (Adolphs, 2009; Hillis, 2014; Jankowski & Takahashi, 2014). These regions could be of key interest as they have been related to mental illness vulnerability and attentional control (Sonuga‐Barke, Cortese, Fairchild, & Stringaris, 2016).

### 4.3 Deprivation exposures

*Deprivation* exposures related to thickness in regions implicated in theorized lack of access to environmental enrichment. Associations specific to thickness support theorized mechanisms of environmentally dependent pruning following synaptic proliferation during late childhood and preadolescence (Changeux & Danchin, 1976; Huttenlocher, de Courten, Garey, & Van der Loos, 1982; Petanjek et al., 2011). Superior frontal thickness predicted objective neighborhood median family income, consistent with the interpretation that exposure to deprivation could accelerate normative developmental synaptic pruning processes (Huttenlocher et al., 1982). Associations with prefrontal thickness are largely consistent with theories of individual‐level deprivation (such as neglect) particularly impacting regions with protracted developmental trajectories (i.e., prefrontal cortex) (McLaughlin et al., 2014; Pechtel & Pizzagalli, 2011; Petanjek et al., 2011). Associations with prefrontal regions are notable given the regions' critical role in neurocognition and executive function (Ronan, Alexander‐Bloch, & Fletcher, 2020; Smolker, Friedman, Hewitt, & Banich, 2018). Current results expand the scope of the theory to systems level deprivation. Notably, *deprivation* exposures identified less related brain regions compared to other domains. Perhaps deprivation exposures share more common features with *stimulation* and *discrepancy* exposures, which was partialled out when controlling for exposure to other domains. Future studies operationalizing deprivation more richly and aiming to replicate current results will help in clarifying possible reasons for the observation.

In addition, as noted earlier, findings highlight the utility of having both self‐report and objective measures across environmental dimensions (as self‐report and objective *deprivation* related to distinct prefrontal regions). Perhaps self‐report *deprivation* picks up on a specific sub‐facet of *deprivation* that neighborhood median family income does not. Future studies are needed to explore this possibility. Even for *discrepancy* and *stimulation* domains, where there was some consistency in associated regions across self‐report and objective measures, there were still regions that uniquely related to self‐report or objective facets of the environmental domain. As such, the notion of subfacets within dimensions warrants further attention and study.

### 4.4 Converging regions across exposures

Taken together, results suggest the three dimensions of systemic environmental exposures relate to specific neural structures, when controlling for other environmental exposures, and for other brain regions. Evidence was also found for common regions predicting multiple types of exposures—this was the case for insula and caudal anterior cingulate area/thickness, which predicted all three domain exposures (though insula did not pass correction for *deprivation*, and caudal anterior cingulate did not pass correction for *discrepancy*). These regions could be implicated through more a general effect of chronic stress exposure (Bauer, 2008; Juster et al., 2010; McEwen, 2017; Vargas et al., 2020). As the insula and caudal anterior cingulate are both implicated in a host of cognitive, affective, and regulatory processes, perhaps exposure during pre‐adolescence marks a sensitive period of neurodevelopment during which development of these regions is particularly malleable (Caruana, Jezzini, Sbriscia‐Fioretti, Rizzolatti, & Gallese, 2011; Eisenberger & Cole, 2012; Uddin, Nomi, Hébert‐Seropian, Ghaziri, & Boucher, 2017). Results support potential regional convergence in neural correlates and exposures. Future investigations are needed to further test converging mechanisms underlying broad, or more general, systemic disadvantage versus specific dimensions and types of exposures.

Further, overall, the magnitude of effects (β) is small according to conventional thresholds. Effect sizes are largely in line with studies of distal neighborhood level characteristics and MRI pooled samples research (Laraia et al., 2012; Lopez, 2007; Sacher et al., 2012). There is reason to believe addressing small individual effects through public health or policy initiatives could have meaningful impacts at the population level (Funder & Ozer, 2019). Once neural correlates of different exposures are better understood, this knowledge could inform the design and content of population‐level targeted prevention or intervention efforts for specific exposures. Future studies could further clarify the effect's significance and relevance to public health initiatives through establishing practical consequences and possible cumulative influences aggregated across development, which would aid in interpreting the effects' ultimate, or practical magnitude. Determining to what extent environmental exposures can impact brain structure, and how pervasive effects may be, is a critical future direction for which longitudinal research is needed. In tandem, developing more granular objective measures of neighborhood and systemic environmental characteristics will be a necessary future step in ascertaining systems level effects.

### 4.5 Broader sample exploratory analyses

The current study was mainly concerned with the top 25th percentile of exposure across domains, and the broader sample had a large proportion of individuals that did not endorse exposure to the environmental domains. Though other environmental exposures were accounted for, the same individuals were not included in all analyses for the high exposure groups, which impairs ability to compare across analyses. For exploratory purposes, analyses with the entire sample are presented in the supplemental material. Of note, while results converged across the entire sample and the high exposure sample, there were also some results that were unique to analyses in the broader sample. Results unique to the broader sample were largely within self‐report measures. Amygdala volume related to both *stimulation* neighborhood safety and *deprivation* neighborhood median family income. Rostral anterior cingulate thickness, which has been found to modulate amygdala‐dependent fear learning, also related to self‐report *deprivation* (Bissière et al., 2008). While the amygdala was a theorized region for the *stimulation* domain, it was not for *deprivation*, highlighting the need for future inquiry and studies examining different developmental periods of exposure.

For *stimulation* domain, neighborhood safety in the broader sample related to lingual thickness and pars orbitalis area, like other visuospatial and semantic processing regions found in the high exposure sample. The lateral orbitofrontal cortex, receiving inputs from visual processing regions, also related to neighborhood safety in the broader sample (Rolls, 2004). Subcortically, the thalamus, critical for perceptual processing, also related to neighborhood safety (Sherman & Guillery, 2006). Finally, while objective neighborhood crimes did not relate to brain morphometry in the high exposure sample, in the broader sample isthmus cingulate thickness, which has been related to stressful life event exposure, related to neighborhood total crimes (Calati et al., 2018).

For *discrepancy* exposures, results largely converged in the broader sample, though there was an association between pars opercularis thickness and *discrepancy* American culture participation, with relations to phonological processing, which had not been theorized. Notably, *deprivation* exposures in the whole sample related to a wider range of regions, including several regions implicated in visual and sensorimotor processing (lateral occipital, precentral, superior parietal, inferior temporal); these results are consistent with theories of deprivation accelerating normative developmental synaptic pruning processes in the human visual cortex (Huttenlocher et al., 1982). Lastly, subcortically, hippocampal and caudate volume related to self‐report deprivation, in line with experience‐dependent plasticity conceptualizations (Kleber, Veit, Birbaumer, Gruzelier, & Lotze, 2010; Wenger & Lövdén, 2016).

### 4.6 SDD theory, limitations, and future directions

The current investigation allowed for an initial test of an emergent conceptual framework, the SDD theory, finding evidence of distinct neural coordinates that remained even after accounting for exposure to other dimensions. The SDD theory proposes that exposure to types of systemic environmental factors can meaningfully aggregate over time and impact neural development across critical sensitive periods (Vargas et al., 2021; Vargas et al., 2020). Further, it poses the notion that teasing apart types, or dimensions, of environmental factors can yield insight to both converging and distinct underlying mechanisms. In a recent study, our group found support for the theory with regards to separating hypothesized *stimulation, discrepancy* and *deprivation* domains, and relating them to mental illness vulnerability (Vargas et al., 2021). The current study tested the neural structures hypothesized to relate to each domain, finding partial support for the theory. While we expected specific neural regions to relate to all three domains, substantial convergence was found for only two out of three domains (for *stimulation* and *discrepancy*). *Deprivation* exposures could engage systems less related to conscious experiences of chronic stress present in *discrepancy* and *stimulation* exposures (Figure S3) (McLaughlin et al., 2021; Nelson III & Gabard‐Durnam, 2020; Takesian & Hensch, 2013). Further study will be needed to modify the conceptual framework.

In addition, while environmental domains related to neural regions that were originally hypothesized by the SDD theory (i.e., cingulate and insular regions for *discrepancy*, prefrontal regions for *deprivation*), there were also a substantial portion of unpredicted findings (see figure 1 in the study by Vargas et al., 2020 for a summary of originally predicted results). For example, *stimulation* and *discrepancy* exposures related to a host of sensory processing regions; this could be due to a neural adaptation geared toward increased attention to environmental cues in the face of increased attentional demands during sensitive developmental periods. Future work is needed to test these interpretations. Contrary to SDD predictions, relations with specific subcortical regions were only observed in the cases of caudate (*stimulation* neighborhood safety), accumbens and hippocampal volume (*discrepancy* neighborhood income inequality). Perhaps subcortical regions such as the hippocampus and amygdala relate to chronic stress more broadly (Bauer, 2008; Juster et al., 2010; McEwen, 2017; Vargas et al., 2020), rather than specifically by one type of exposure over and above the others. In addition, *Stimulation* exposures related to prefrontal regions beyond what was originally theorized, including precentral and paracentral regions. Observed results could also be due to developmental timing; perhaps exposure relates more to prefrontal morphology and subcortical structures earlier or later in development. Future studies are needed to clarify. In addition, self‐report and objective measures of *deprivation* did not share overlapping neural regions. Future study is needed to further understand relations between self‐report and objective measures, as well as underlying mechanisms for each domain.

With regards to the expected co‐occurrence of multiple environmental exposures within individuals, co‐occurrence of exposures was not a major concern, as strong associations did not emerge between environmental dimensions in most cases, except for analyses on the high population density subsample. Given the high association between neighborhood crime and population density in that set of analyses, results ought to be taken as preliminary and interpreted with caution—future investigations will be needed to determine whether observed relations are generalizable to geographic locations beyond the current sample. More broadly, it will be crucial for future investigations to identify and account for confounding factors, including manners unrelated to the questions of interest in which high exposure environments could be systematically different from low‐exposure environments. The current study is informative in bringing attention to how systemic, contextual environmental factors could relate to gray matter morphometry. The work adds to existing insights from ABCD data, which have often explored single features of systemic environmental factors (Mullins et al., 2020; Taylor et al., 2020; Vargas et al., 2020).

The current work highlights the promise of incorporating multiple types of exposures within the same sample for understanding candidate mechanisms. However, future studies teasing apart mechanisms through which systemic environmental exposures could be impactful will be crucial. Previous ABCD studies have established the general effects of environmental disadvantage on overall gray matter morphometry (Karcher et al., 2021). Future studies could build on risk conceptualizations by assessing relations to stress, as well as separating effects due to stress from effects due to experience dependent plasticity, or due to altered neurodevelopment more broadly (McLaughlin et al., 2021). In addition, systemic factors have received limited attention in the literature and work exploring these factors is necessary to build a robust foundation of knowledge. The current study sought to contribute to these efforts. Future investigations will benefit from measuring both systemic environmental factors as well as more proximal individual‐level exposures and examining interactions and dynamic processes between the two. Indeed, early foundational work in this area has already begun to show that accounting for both individual and systemic factors is necessary (Tomasi & Volkow, 2021).

Investigating systemic environmental exposures, as this study sought to do, is a contributing step toward understanding systemic inequalities in the United States. To better understand existing disparities in health and well‐being among marginalized groups, it is key to identify influencing factors. The SDD theory aims to identify dimensions of environmental exposures that could systemically confer vulnerability for adverse health and functional outcomes. The sequalae of systemic bias, inequalities, and disadvantage resulting in racial disparities and disparities among marginalized groups is complex and multifaceted. To fully conceptualize these problems, it is essential to account for proximal factors, as well as for systemic/contextual/structural environmental factors across multiple levels. What is more, identifying mechanisms through which disadvantage could manifest is necessary for any future system‐level or policy efforts geared toward intervention and prevention of systemic inequities. The current study hopes to contribute to this pursuit by identifying putative neural correlates of dimensions of systemic exposures based on prior literature.

As mentioned, one of the strengths of the study lies in investigating environmental factors during a dynamic period of neurodevelopment: pre‐adolescence (Jeon et al., 2015; Lyall et al., 2015; Tamnes et al., 2017; Wierenga et al., 2014). Foundational processes readying the organism for puberty (including adrenarche and gonadarche), along with widespread pruning and specialization, make this stage an impactful period to understand (Mills, Lalonde, Clasen, Giedd, & Blakemore, 2014). With the developmental timing of thickness and surface area varying by cortical region, and cortical thinning and pruning processes being highly active during this time, there is marked neural reorganization occurring. As such, this period is prime for assessing for plasticity, or sensitivity to environmental influences (Nelson & Gabard‐Durnam, 2020; Pechtel & Pizzagalli, 2011). Future studies will be crucial in determining effects of neurodevelopmental stage on observed associations, as these may not generalize to other developmental stages. By the same token, it is necessary to contextualize results in the developmental stage that they were observed: pre‐adolescence.

Future studies would also benefit from incorporating multiple time points to better infer possible mechanisms of influence and incorporate multiple developmental sensitive periods. Given that exploring relations to biological sex was outside the scope of the current study, sex was controlled for in all analyses. However, future studies would benefit from investigating sex‐specific relationships, given the key developmental of prepuberty, which includes several biological and neurological sequelae that differ based on biological sex (Mills et al., 2014). Future investigations could also build on this early work by exploring questions related to hemisphere asymmetry and laterality. Expanding beyond gray matter morphometry to functional imaging and white matter would also enrich understanding of possible underlying mechanisms of influence. Further teasing out functional and symptom outcomes relating to the exposures is also a crucial future direction. In all, the present study offers a first step toward unmasking neural correlates of systemic environmental exposures, which ultimately could inform public health policy, prevention and intervention efforts for vulnerable populations.

## CONFLICTS OF INTEREST

The authors declare no conflicts of interest in relation to the subject of the study.

## ETHICS STATEMENT

All procedures were approved by local institutional review boards.

## PATIENT CONSENT

Participants provided informed consent.

## Supporting information


**Appendix S1**: Supporting Information

## Data Availability

Data sharing and data accessibility: data is available through the ABCD data repository at nda.nih.gov.
