# Supplementary material for: Differentiating distinct and converging neural correlates of types of systemic environmental exposures
Source: Hum Brain Mapp. 2022 Jan 22;43(7):2232–48. doi: 10.1002/hbm.25783 (PMC8996350; doi:10.1002/hbm.25783)
Supplement: Supplementary file 1 — Appendix S1: Supporting Information [file HBM-43-2232-s001.docx]

**Supplementary material**

**Supplementary table 1**

*Individual items comprising the self-report factors, for more information see (Vargas et al., 2021).*

| Self-report domain | Item full prompt |
| --- | --- |
| Discrepancy/sense of belonging with ethnic group  (Likert scale 1-5, 1=Strongly disagree, 5=Strongly agree) | I have spent time trying to find out more about my ethnic group, such as its history, traditions, and customs. |
|  | I have a strong sense of belonging to my own ethnic group. |
|  | I understand pretty well what my ethnic group membership means to me. |
|  | I have often done things that will help me understand my ethnic background better. |
|  | I have often talked to other people in order to learn more about my ethnic group. |
|  | I feel a strong attachment towards my own ethnic group. |
| Discrepancy/American culture participation (Likert scale 1-9, 1=Disagree, 9=Agree) | I often participate in mainstream American cultural traditions. |
|  | I enjoy social activities with typical American people. |
|  | I am comfortable interacting with typical American people. |
|  | I enjoy typical American entertainment (e.g. movies, music). |
|  | I often behave in ways that are typically American. |
|  | It is important for me to maintain or develop American mainstream cultural practices. |
|  | I believe in mainstream American values. |
|  | I am interested in having typical American friends. |
| Stimulation/neighborhood safety  (Likert scale 1-5, 1=Strongly disagree, 5=Strongly agree) | I feel safe walking in my neighborhood, day or night. |
|  | Violence is not a problem in my neighborhood. |
|  | My neighborhood is safe from crime. |
| Deprivation/Lack of access to financial, health, or nutritional resources  (Dichotomous scale 0-1, 0=has not occurred, 1=has occurred) | In the past 12 months, you and your immediate family: Needed food but couldn't afford to buy it or couldn't afford to go out to get it? |
|  | In the past 12 months, you and your immediate family: Were without telephone service because you could not afford it? |
|  | In the past 12 months, you and your immediate family: Didn't pay the full amount of the rent or mortgage because you could not afford it? |
|  | In the past 12 months, you and your immediate family: Had services turned off by the gas or electric company, or the oil company wouldn't deliver oil because payments were not made? |
|  | In the past 12 months, you and your immediate family: Had someone who needed to see a doctor or go to the hospital but didn't go because you could not afford it? |
|  | In the past 12 months, you and your immediate family: Had someone who needed a dentist but couldn't go because you could not afford it? |

**Supplementary table 2**

*Cortical thickness and area regions predicting discrepancy/sense of belonging with ethnic group self-report factor, accounting for stimulation and deprivation self-report factors, age and sex as fixed effects, with family and scanner as a random effect, SE=standard error, β=standardized β coefficient, ✓ denotes that result passed Bonferroni correction threshold of p<0.0167.*

|  | Cortical thickness | | | |  | Cortical area | | | |  |
| --- | --- | --- | --- | --- | --- | --- | --- | --- | --- | --- |
|  | β | SE | t-value | p-value | Pass Bonferroni? | β | SE | t-value | p-value | Pass Bonferroni? |
| Intercept | -0.015 | 0.029 | -0.530 | 0.596 |  | -0.013 | 0.029 | -0.448 | 0.654 |  |
| Banks of superior temporal sulcus | -0.008 | 0.023 | -0.331 | 0.741 |  | 0.028 | 0.024 | 1.157 | 0.249 |  |
| Caudal anterior cingulate | 0.001 | 0.020 | 0.046 | 0.964 |  | -0.007 | 0.026 | -0.282 | 0.778 |  |
| Caudal middle frontal | -0.034 | 0.030 | -1.112 | 0.267 |  | -0.003 | 0.026 | -0.102 | 0.919 |  |
| Cuneus | -0.021 | 0.025 | -0.827 | 0.409 |  | 0.062 | 0.030 | 2.108 | 0.036 |  |
| Entorhinal | 0.032 | 0.019 | 1.651 | 0.100 |  | 0.010 | 0.021 | 0.491 | 0.624 |  |
| Fusiform | -0.004 | 0.029 | -0.132 | 0.895 |  | -0.013 | 0.030 | -0.427 | 0.670 |  |
| Inferior parietal | -0.011 | 0.038 | -0.305 | 0.760 |  | -0.036 | 0.029 | -1.223 | 0.223 |  |
| Inferior temporal | 0.003 | 0.028 | 0.120 | 0.904 |  | 0.053 | 0.034 | 1.546 | 0.124 |  |
| Isthmus cingulate | -0.002 | 0.018 | -0.119 | 0.906 |  | 0.011 | 0.027 | 0.418 | 0.676 |  |
| Lateral occipital | 0.049 | 0.033 | 1.487 | 0.139 |  | -0.024 | 0.028 | -0.857 | 0.392 |  |
| Lateral orbitofrontal | 0.034 | 0.026 | 1.300 | 0.195 |  | -0.002 | 0.032 | -0.074 | 0.941 |  |
| Lingual | 0.036 | 0.025 | 1.424 | 0.156 |  | 0.016 | 0.030 | 0.528 | 0.598 |  |
| Medial orbitofrontal | -0.041 | 0.024 | -1.724 | 0.086 |  | -0.026 | 0.030 | -0.879 | 0.381 |  |
| Middle temporal | -0.009 | 0.031 | -0.300 | 0.765 |  | -0.025 | 0.037 | -0.669 | 0.504 |  |
| Parahippocampal | 0.010 | 0.018 | 0.571 | 0.568 |  | 0.004 | 0.020 | 0.190 | 0.850 |  |
| Paracentral | -0.008 | 0.028 | -0.298 | 0.766 |  | 0.040 | 0.024 | 1.676 | 0.095 |  |
| Pars opercularis | 0.044 | 0.025 | 1.726 | 0.086 |  | -0.013 | 0.024 | -0.562 | 0.575 |  |
| Pars orbitalis | -0.028 | 0.022 | -1.252 | 0.212 |  | 0.004 | 0.028 | 0.136 | 0.892 |  |
| Parstriangularis | 0.014 | 0.028 | 0.503 | 0.616 |  | 0.017 | 0.024 | 0.698 | 0.486 |  |
| Pericalcarine | -0.045 | 0.024 | -1.886 | 0.061 |  | -0.055 | 0.033 | -1.689 | 0.093 |  |
| Postcentral | -0.020 | 0.028 | -0.721 | 0.472 |  | -0.009 | 0.031 | -0.287 | 0.774 |  |
| Posterior cingulate | 0.030 | 0.021 | 1.396 | 0.164 |  | -0.017 | 0.026 | -0.661 | 0.509 |  |
| Precentral | 0.019 | 0.032 | 0.592 | 0.554 |  | 0.007 | 0.029 | 0.254 | 0.800 |  |
| Precuneus | 0.016 | 0.030 | 0.551 | 0.582 |  | -0.002 | 0.031 | -0.050 | 0.960 |  |
| Rostral anterior cingulate | 0.038 | 0.020 | 1.876 | 0.062 |  | 0.039 | 0.028 | 1.418 | 0.158 |  |
| Rostral middle frontal | -0.033 | 0.037 | -0.896 | 0.371 |  | 0.036 | 0.033 | 1.087 | 0.278 |  |
| Superior frontal | 0.002 | 0.035 | 0.043 | 0.965 |  | -0.013 | 0.041 | -0.326 | 0.745 |  |
| Superior parietal | -0.007 | 0.037 | -0.181 | 0.857 |  | -0.021 | 0.029 | -0.739 | 0.461 |  |
| Superior temporal | -0.013 | 0.031 | -0.425 | 0.671 |  | -0.012 | 0.038 | -0.302 | 0.763 |  |
| Supramarginal | 0.009 | 0.036 | 0.249 | 0.804 |  | -0.037 | 0.031 | -1.193 | 0.234 |  |
| Frontal pole | 0.008 | 0.020 | 0.397 | 0.692 |  | 0.002 | 0.022 | 0.102 | 0.919 |  |
| Temporal pole | -0.029 | 0.019 | -1.500 | 0.135 |  | 0.027 | 0.022 | 1.215 | 0.226 |  |
| Transverse temporal | -0.011 | 0.021 | -0.518 | 0.605 |  | 0.002 | 0.024 | 0.075 | 0.940 |  |
| Insula | -0.071 | 0.023 | -3.143 | 0.002 | ✓ | 0.000 | 0.028 | -0.013 | 0.990 |  |
| Stimulation factor | -0.015 | 0.021 | -0.727 | 0.468 |  | -0.014 | 0.021 | -0.680 | 0.497 |  |
| Deprivation factor | -0.001 | 0.023 | -0.038 | 0.970 |  | 0.002 | 0.023 | 0.104 | 0.918 |  |
| Discrepancy/American culture participation factor | 0.152 | 0.022 | 6.815 | 0.000 |  | 0.153 | 0.022 | 6.855 | 0.000 |  |
| Age | 0.026 | 0.016 | 1.641 | 0.102 |  | 0.031 | 0.015 | 2.046 | 0.042 |  |
| Sex | 0.018 | 0.019 | 0.975 | 0.331 |  | 0.004 | 0.021 | 0.190 | 0.849 |  |

**Supplementary table 3**

*Cortical thickness and area regions predicting stimulation/neighborhood safety self-report factor, accounting for discrepancy and deprivation self-report factors, age and sex as fixed effects with family and scanner as a random effect, SE=standard error, β=standardized β coefficient, ✓ denotes that result passed Bonferroni correction threshold of p<0.0167.*

|  | Cortical thickness | | | |  | Cortical area | | | |  |
| --- | --- | --- | --- | --- | --- | --- | --- | --- | --- | --- |
|  | β | SE | t-value | p-value | Pass Bonferroni? | β | SE | t-value | p-value | Pass Bonferroni? |
| Intercept | 0.034 | 0.039 | 0.887 | 0.375 |  | 0.036 | 0.041 | 0.880 | 0.379 |  |
| Banks of superior temporal sulcus | 0.038 | 0.029 | 1.317 | 0.190 |  | -0.021 | 0.031 | -0.664 | 0.508 |  |
| Caudal anterior cingulate | -0.058 | 0.024 | -2.380 | 0.019 |  | 0.028 | 0.032 | 0.881 | 0.380 |  |
| Caudal middle frontal | 0.075 | 0.037 | 2.041 | 0.044 |  | -0.014 | 0.031 | -0.456 | 0.649 |  |
| Cuneus | -0.024 | 0.035 | -0.681 | 0.497 |  | 0.020 | 0.037 | 0.523 | 0.602 |  |
| Entorhinal | 0.012 | 0.024 | 0.510 | 0.611 |  | 0.020 | 0.026 | 0.760 | 0.449 |  |
| Fusiform | 0.002 | 0.037 | 0.058 | 0.954 |  | 0.026 | 0.037 | 0.722 | 0.472 |  |
| Inferior parietal | -0.030 | 0.049 | -0.608 | 0.545 |  | -0.046 | 0.036 | -1.277 | 0.204 |  |
| Inferior temporal | 0.006 | 0.035 | 0.179 | 0.858 |  | -0.002 | 0.042 | -0.038 | 0.969 |  |
| Isthmus cingulate | -0.017 | 0.023 | -0.756 | 0.451 |  | -0.038 | 0.031 | -1.228 | 0.222 |  |
| Lateral occipital | 0.034 | 0.041 | 0.820 | 0.414 |  | 0.004 | 0.034 | 0.115 | 0.909 |  |
| Lateral orbitofrontal | 0.051 | 0.034 | 1.499 | 0.137 |  | 0.005 | 0.040 | 0.136 | 0.892 |  |
| Lingual | 0.051 | 0.032 | 1.566 | 0.120 |  | -0.015 | 0.036 | -0.419 | 0.676 |  |
| Medial orbitofrontal | -0.033 | 0.031 | -1.055 | 0.294 |  | 0.022 | 0.035 | 0.633 | 0.528 |  |
| Middle temporal | 0.006 | 0.040 | 0.151 | 0.880 |  | 0.039 | 0.047 | 0.833 | 0.407 |  |
| Parahippocampal | 0.003 | 0.023 | 0.140 | 0.889 |  | 0.026 | 0.027 | 0.963 | 0.338 |  |
| Paracentral | -0.023 | 0.035 | -0.645 | 0.520 |  | -0.019 | 0.029 | -0.656 | 0.514 |  |
| Pars opercularis | 0.006 | 0.033 | 0.168 | 0.867 |  | 0.057 | 0.030 | 1.898 | 0.060 |  |
| Pars orbitalis | -0.031 | 0.029 | -1.058 | 0.292 |  | -0.003 | 0.033 | -0.103 | 0.918 |  |
| Parstriangularis | -0.013 | 0.034 | -0.374 | 0.709 |  | 0.025 | 0.029 | 0.886 | 0.378 |  |
| Pericalcarine | -0.001 | 0.029 | -0.045 | 0.964 |  | -0.016 | 0.039 | -0.403 | 0.688 |  |
| Postcentral | -0.011 | 0.033 | -0.335 | 0.738 |  | 0.029 | 0.036 | 0.805 | 0.423 |  |
| Posterior cingulate | 0.012 | 0.025 | 0.474 | 0.637 |  | -0.028 | 0.032 | -0.857 | 0.393 |  |
| Precentral | 0.048 | 0.038 | 1.255 | 0.212 |  | -0.024 | 0.035 | -0.679 | 0.499 |  |
| Precuneus | 0.044 | 0.040 | 1.103 | 0.273 |  | 0.010 | 0.037 | 0.273 | 0.786 |  |
| Rostral anterior cingulate | 0.044 | 0.026 | 1.697 | 0.093 |  | -0.030 | 0.034 | -0.887 | 0.377 |  |
| Rostral middle frontal | 0.028 | 0.046 | 0.619 | 0.537 |  | 0.020 | 0.040 | 0.509 | 0.612 |  |
| Superior frontal | -0.054 | 0.045 | -1.199 | 0.233 |  | 0.007 | 0.047 | 0.145 | 0.885 |  |
| Superior parietal | 0.004 | 0.048 | 0.081 | 0.935 |  | 0.000 | 0.035 | 0.010 | 0.992 |  |
| Superior temporal | -0.004 | 0.039 | -0.106 | 0.916 |  | 0.054 | 0.046 | 1.175 | 0.243 |  |
| Supramarginal | -0.068 | 0.046 | -1.497 | 0.137 |  | 0.006 | 0.035 | 0.176 | 0.860 |  |
| Frontal pole | -0.019 | 0.025 | -0.762 | 0.448 |  | -0.029 | 0.026 | -1.129 | 0.261 |  |
| Temporal pole | -0.067 | 0.025 | -2.621 | 0.010 | ✓ | 0.037 | 0.026 | 1.442 | 0.152 |  |
| Transverse temporal | 0.018 | 0.028 | 0.640 | 0.523 |  | -0.086 | 0.031 | -2.772 | 0.007 | ✓ |
| Insula | -0.025 | 0.028 | -0.908 | 0.366 |  | -0.036 | 0.035 | -1.047 | 0.297 |  |
| Discrepancy/American culture participation | 0.053 | 0.023 | 2.322 | 0.022 |  | 0.046 | 0.023 | 2.036 | 0.044 |  |
| Deprivation/deprivation | -0.145 | 0.022 | -6.510 | 0.000 |  | -0.144 | 0.022 | -6.458 | 0.000 |  |
| Discrepancy/Sense of belonging with ethnic group | -0.024 | 0.023 | -1.083 | 0.281 |  | -0.024 | 0.022 | -1.050 | 0.296 |  |
| Age | -0.006 | 0.021 | -0.280 | 0.780 |  | -0.020 | 0.020 | -0.996 | 0.321 |  |
| Sex | 0.016 | 0.023 | 0.705 | 0.482 |  | 0.008 | 0.025 | 0.326 | 0.745 |  |

**Supplementary table 4**

*Cortical thickness and area regions predicting deprivation/deprivation self-report factor, accounting for discrepancy and deprivation self-report factors, age and sex as fixed effects, with family and scanner as a random effect, SE=standard error, β=standardized β coefficient, ✓ denotes that result passed Bonferroni correction threshold of p<0.0167.*

|  | Cortical thickness | | | |  | Cortical area | | | |  |
| --- | --- | --- | --- | --- | --- | --- | --- | --- | --- | --- |
|  | β | SE | t-value | p-value | Pass Bonferroni? | β | SE | t-value | p-value | Pass Bonferroni? |
| Intercept | -0.016 | 0.046 | -0.351 | 0.726 |  | -0.011 | 0.046 | -0.242 | 0.809 |  |
| Banks of superior temporal sulcus | 0.017 | 0.030 | 0.583 | 0.561 |  | 0.043 | 0.029 | 1.481 | 0.141 |  |
| Caudal anterior cingulate | -0.037 | 0.024 | -1.551 | 0.124 |  | -0.076 | 0.031 | -2.457 | 0.015 | ✓ |
| Caudal middle frontal | 0.057 | 0.036 | 1.586 | 0.115 |  | -0.020 | 0.031 | -0.656 | 0.513 |  |
| Cuneus | -0.044 | 0.032 | -1.396 | 0.165 |  | 0.002 | 0.036 | 0.067 | 0.947 |  |
| Entorhinal | 0.012 | 0.024 | 0.498 | 0.620 |  | 0.007 | 0.025 | 0.290 | 0.772 |  |
| Fusiform | 0.000 | 0.033 | 0.004 | 0.997 |  | 0.048 | 0.036 | 1.329 | 0.186 |  |
| Inferior parietal | 0.004 | 0.046 | 0.077 | 0.938 |  | -0.011 | 0.035 | -0.297 | 0.767 |  |
| Inferior temporal | -0.045 | 0.034 | -1.301 | 0.196 |  | -0.054 | 0.040 | -1.358 | 0.177 |  |
| Isthmus cingulate | -0.006 | 0.023 | -0.235 | 0.815 |  | 0.013 | 0.032 | 0.402 | 0.688 |  |
| Lateral occipital | -0.034 | 0.039 | -0.868 | 0.387 |  | -0.047 | 0.034 | -1.374 | 0.172 |  |
| Lateral orbitofrontal | 0.015 | 0.030 | 0.521 | 0.603 |  | 0.008 | 0.039 | 0.193 | 0.848 |  |
| Lingual | 0.011 | 0.032 | 0.334 | 0.739 |  | -0.049 | 0.035 | -1.425 | 0.157 |  |
| Medial orbitofrontal | 0.053 | 0.030 | 1.770 | 0.079 |  | -0.014 | 0.034 | -0.414 | 0.680 |  |
| Middle temporal | 0.019 | 0.041 | 0.458 | 0.648 |  | 0.038 | 0.043 | 0.866 | 0.388 |  |
| Parahippocampal | 0.004 | 0.024 | 0.185 | 0.854 |  | 0.007 | 0.025 | 0.301 | 0.764 |  |
| Paracentral | 0.008 | 0.034 | 0.235 | 0.814 |  | -0.014 | 0.026 | -0.547 | 0.585 |  |
| Pars opercularis | -0.049 | 0.033 | -1.492 | 0.138 |  | 0.005 | 0.027 | 0.201 | 0.841 |  |
| Pars orbitalis | 0.013 | 0.027 | 0.463 | 0.644 |  | -0.017 | 0.031 | -0.540 | 0.590 |  |
| Parstriangularis | -0.017 | 0.032 | -0.534 | 0.594 |  | -0.033 | 0.028 | -1.188 | 0.237 |  |
| Pericalcarine | 0.005 | 0.030 | 0.173 | 0.863 |  | 0.061 | 0.037 | 1.636 | 0.104 |  |
| Postcentral | -0.015 | 0.032 | -0.471 | 0.639 |  | 0.019 | 0.034 | 0.573 | 0.568 |  |
| Posterior cingulate | 0.033 | 0.026 | 1.243 | 0.217 |  | -0.045 | 0.032 | -1.388 | 0.168 |  |
| Precentral | -0.054 | 0.036 | -1.489 | 0.139 |  | 0.024 | 0.035 | 0.687 | 0.493 |  |
| Precuneus | 0.047 | 0.037 | 1.257 | 0.211 |  | 0.003 | 0.037 | 0.075 | 0.940 |  |
| Rostral anterior cingulate | 0.017 | 0.024 | 0.699 | 0.486 |  | 0.020 | 0.032 | 0.631 | 0.529 |  |
| Rostral middle frontal | 0.029 | 0.045 | 0.631 | 0.529 |  | 0.023 | 0.036 | 0.642 | 0.522 |  |
| Superior frontal | -0.018 | 0.045 | -0.401 | 0.689 |  | -0.072 | 0.047 | -1.551 | 0.124 |  |
| Superior parietal | -0.062 | 0.048 | -1.307 | 0.194 |  | -0.034 | 0.036 | -0.962 | 0.338 |  |
| Superior temporal | 0.025 | 0.040 | 0.627 | 0.532 |  | 0.042 | 0.046 | 0.916 | 0.362 |  |
| Supramarginal | 0.071 | 0.045 | 1.566 | 0.120 |  | -0.006 | 0.033 | -0.173 | 0.863 |  |
| Frontal pole | -0.004 | 0.025 | -0.181 | 0.857 |  | -0.019 | 0.024 | -0.798 | 0.426 |  |
| Temporal pole | -0.006 | 0.025 | -0.238 | 0.812 |  | 0.020 | 0.026 | 0.782 | 0.436 |  |
| Transverse temporal | 0.009 | 0.027 | 0.324 | 0.747 |  | -0.039 | 0.029 | -1.329 | 0.186 |  |
| Insula | -0.045 | 0.027 | -1.658 | 0.100 |  | 0.048 | 0.034 | 1.431 | 0.155 |  |
| Stimulation/neighborhood safety | -0.104 | 0.023 | -4.458 | 0.000 |  | -0.101 | 0.023 | -4.400 | 0.000 |  |
| Discrepancy/American culture participation | -0.055 | 0.027 | -2.015 | 0.046 |  | -0.052 | 0.027 | -1.913 | 0.058 |  |
| Discrepancy/Sense of belonging with ethnic group | 0.019 | 0.026 | 0.726 | 0.469 |  | 0.021 | 0.026 | 0.820 | 0.414 |  |
| Age | 0.037 | 0.020 | 1.863 | 0.065 |  | 0.036 | 0.018 | 1.958 | 0.053 |  |
| Sex | 0.037 | 0.023 | 1.587 | 0.115 |  | 0.058 | 0.025 | 2.289 | 0.024 |  |

**Supplementary table 5**

*Cortical thickness and area regions predicting discrepancy/American culture participation self-report factor, accounting for stimulation and deprivation self-report factors, age and sex as fixed effects, with family and scanner as a random effect, SE=standard error, β=standardized β coefficient, ✓ denotes that result passed Bonferroni correction threshold of p<0.0167.*

|  | Cortical thickness | | | |  | Cortical area | | | |  |
| --- | --- | --- | --- | --- | --- | --- | --- | --- | --- | --- |
|  | β | SE | t-value | p-value | Pass Bonferroni? | β | SE | t-value | p-value | Pass Bonferroni? |
| Intercept | 0.015 | 0.037 | 0.399 | 0.690 |  | 0.010 | 0.038 | 0.279 | 0.780 |  |
| Banks of superior temporal sulcus | -0.017 | 0.023 | -0.749 | 0.455 |  | -0.022 | 0.022 | -1.001 | 0.318 |  |
| Caudal anterior cingulate | -0.028 | 0.019 | -1.467 | 0.144 |  | 0.005 | 0.023 | 0.208 | 0.835 |  |
| Caudal middle frontal | -0.030 | 0.029 | -1.026 | 0.306 |  | 0.036 | 0.023 | 1.581 | 0.116 |  |
| Cuneus | 0.016 | 0.025 | 0.636 | 0.526 |  | -0.018 | 0.028 | -0.635 | 0.527 |  |
| Entorhinal | -0.006 | 0.019 | -0.329 | 0.743 |  | -0.013 | 0.019 | -0.691 | 0.491 |  |
| Fusiform | -0.010 | 0.027 | -0.349 | 0.728 |  | 0.012 | 0.026 | 0.439 | 0.661 |  |
| Inferior parietal | 0.088 | 0.036 | 2.470 | 0.015 | ✓ | -0.047 | 0.026 | -1.797 | 0.074 |  |
| Inferior temporal | -0.042 | 0.026 | -1.612 | 0.109 |  | -0.050 | 0.029 | -1.701 | 0.091 |  |
| Isthmus cingulate | 0.010 | 0.019 | 0.532 | 0.596 |  | -0.073 | 0.024 | -2.991 | 0.003 | ✓ |
| Lateral occipital | 0.021 | 0.033 | 0.644 | 0.520 |  | 0.038 | 0.026 | 1.441 | 0.152 |  |
| Lateral orbitofrontal | -0.004 | 0.025 | -0.175 | 0.862 |  | 0.029 | 0.030 | 0.947 | 0.345 |  |
| Lingual | 0.001 | 0.024 | 0.024 | 0.981 |  | -0.013 | 0.027 | -0.470 | 0.639 |  |
| Medial orbitofrontal | -0.017 | 0.024 | -0.707 | 0.481 |  | -0.023 | 0.027 | -0.862 | 0.390 |  |
| Middle temporal | 0.000 | 0.031 | -0.007 | 0.995 |  | 0.066 | 0.033 | 1.976 | 0.050 |  |
| Parahippocampal | 0.018 | 0.018 | 1.048 | 0.296 |  | 0.005 | 0.020 | 0.229 | 0.819 |  |
| Paracentral | -0.016 | 0.026 | -0.625 | 0.533 |  | -0.057 | 0.022 | -2.595 | 0.010 | ✓ |
| Pars opercularis | -0.034 | 0.024 | -1.400 | 0.164 |  | -0.025 | 0.020 | -1.229 | 0.221 |  |
| Pars orbitalis | -0.013 | 0.020 | -0.638 | 0.524 |  | 0.018 | 0.025 | 0.717 | 0.474 |  |
| Parstriangularis | 0.014 | 0.025 | 0.551 | 0.582 |  | -0.016 | 0.021 | -0.754 | 0.452 |  |
| Pericalcarine | -0.030 | 0.023 | -1.343 | 0.181 |  | 0.016 | 0.030 | 0.526 | 0.600 |  |
| Postcentral | -0.017 | 0.027 | -0.648 | 0.518 |  | -0.032 | 0.027 | -1.159 | 0.248 |  |
| Posterior cingulate | -0.006 | 0.020 | -0.297 | 0.767 |  | 0.036 | 0.025 | 1.412 | 0.160 |  |
| Precentral | -0.012 | 0.030 | -0.404 | 0.687 |  | 0.079 | 0.025 | 3.132 | 0.002 | ✓ |
| Precuneus | -0.009 | 0.030 | -0.294 | 0.769 |  | 0.025 | 0.027 | 0.928 | 0.355 |  |
| Rostral anterior cingulate | 0.004 | 0.019 | 0.185 | 0.853 |  | -0.029 | 0.025 | -1.156 | 0.250 |  |
| Rostral middle frontal | 0.033 | 0.035 | 0.933 | 0.353 |  | 0.003 | 0.029 | 0.108 | 0.914 |  |
| Superior frontal | -0.008 | 0.034 | -0.230 | 0.819 |  | 0.038 | 0.036 | 1.038 | 0.301 |  |
| Superior parietal | 0.015 | 0.034 | 0.433 | 0.665 |  | 0.004 | 0.026 | 0.160 | 0.873 |  |
| Superior temporal | 0.031 | 0.031 | 1.006 | 0.316 |  | -0.017 | 0.035 | -0.482 | 0.630 |  |
| Supramarginal | 0.001 | 0.035 | 0.033 | 0.974 |  | 0.013 | 0.027 | 0.465 | 0.643 |  |
| Frontal pole | 0.007 | 0.019 | 0.384 | 0.701 |  | 0.019 | 0.019 | 0.992 | 0.323 |  |
| Temporal pole | -0.014 | 0.018 | -0.771 | 0.442 |  | 0.016 | 0.019 | 0.820 | 0.413 |  |
| Transverse temporal | 0.018 | 0.021 | 0.863 | 0.389 |  | 0.008 | 0.022 | 0.365 | 0.716 |  |
| Insula | 0.044 | 0.020 | 2.158 | 0.032 |  | -0.021 | 0.028 | -0.765 | 0.445 |  |
| Stimulation/neighborhood safety | 0.063 | 0.020 | 3.140 | 0.002 |  | 0.055 | 0.020 | 2.830 | 0.005 |  |
| Deprivation/deprivation | -0.071 | 0.023 | -3.112 | 0.002 |  | -0.065 | 0.023 | -2.875 | 0.005 |  |
| Discrepancy/sense of belonging | 0.168 | 0.022 | 7.744 | 0.000 |  | 0.173 | 0.021 | 8.069 | 0.000 |  |
| Age | 0.006 | 0.014 | 0.390 | 0.697 |  | -0.002 | 0.013 | -0.171 | 0.864 |  |
| Sex | 0.009 | 0.018 | 0.510 | 0.611 |  | 0.003 | 0.020 | 0.147 | 0.883 |  |

**Supplementary table 6**

*Cortical thickness and area regions predicting deprivation/neighborhood median family income objective measure, accounting for stimulation and discrepancy objective factors, age and sex as fixed effects, with family and scanner as a random effect, SE=standard error, β=standardized β coefficient, ✓ denotes that result passed Bonferroni correction threshold of p<0.0167.*

|  | Cortical thickness | | | |  | Cortical area | | | |  |
| --- | --- | --- | --- | --- | --- | --- | --- | --- | --- | --- |
|  | β | SE | t-value | p-value | Pass Bonferroni? | β | SE | t-value | p-value | Pass Bonferroni? |
| Intercept | 0.033 | 0.038 | 0.849 | 0.396 |  | 0.030 | 0.039 | 0.762 | 0.446 |  |
| Banks of superior temporal sulcus | -0.001 | 0.012 | -0.042 | 0.966 |  | -0.010 | 0.013 | -0.750 | 0.454 |  |
| Caudal anterior cingulate | 0.004 | 0.010 | 0.339 | 0.735 |  | 0.009 | 0.013 | 0.724 | 0.470 |  |
| Caudal middle frontal | 0.009 | 0.017 | 0.519 | 0.604 |  | 0.007 | 0.013 | 0.583 | 0.561 |  |
| Cuneus | -0.015 | 0.014 | -1.102 | 0.272 |  | 0.015 | 0.015 | 1.057 | 0.292 |  |
| Entorhinal | 0.005 | 0.010 | 0.444 | 0.657 |  | 0.005 | 0.010 | 0.488 | 0.626 |  |
| Fusiform | 0.015 | 0.015 | 0.975 | 0.331 |  | 0.005 | 0.015 | 0.352 | 0.725 |  |
| Inferior parietal | 0.002 | 0.022 | 0.082 | 0.935 |  | 0.020 | 0.015 | 1.370 | 0.172 |  |
| Inferior temporal | 0.008 | 0.015 | 0.527 | 0.599 |  | 0.019 | 0.018 | 1.063 | 0.289 |  |
| Isthmus cingulate | -0.008 | 0.010 | -0.834 | 0.405 |  | 0.004 | 0.014 | 0.265 | 0.792 |  |
| Lateral occipital | -0.014 | 0.018 | -0.776 | 0.439 |  | -0.003 | 0.014 | -0.199 | 0.843 |  |
| Lateral orbitofrontal | 0.009 | 0.014 | 0.635 | 0.526 |  | 0.014 | 0.017 | 0.844 | 0.400 |  |
| Lingual | 0.015 | 0.014 | 1.101 | 0.272 |  | -0.017 | 0.015 | -1.120 | 0.264 |  |
| Medial orbitofrontal | -0.026 | 0.013 | -2.091 | 0.038 |  | 0.002 | 0.014 | 0.167 | 0.868 |  |
| Middle temporal | 0.007 | 0.018 | 0.373 | 0.710 |  | -0.002 | 0.019 | -0.082 | 0.935 |  |
| Parahippocampal | 0.005 | 0.010 | 0.514 | 0.608 |  | 0.009 | 0.011 | 0.805 | 0.422 |  |
| Paracentral | 0.009 | 0.015 | 0.581 | 0.562 |  | 0.010 | 0.012 | 0.786 | 0.433 |  |
| Pars opercularis | 0.007 | 0.014 | 0.530 | 0.597 |  | -0.001 | 0.012 | -0.052 | 0.959 |  |
| Pars orbitalis | 0.022 | 0.012 | 1.799 | 0.074 |  | -0.019 | 0.013 | -1.483 | 0.140 |  |
| Parstriangularis | -0.001 | 0.014 | -0.074 | 0.941 |  | 0.015 | 0.012 | 1.277 | 0.203 |  |
| Pericalcarine | 0.010 | 0.012 | 0.854 | 0.394 |  | -0.018 | 0.016 | -1.142 | 0.255 |  |
| Postcentral | -0.002 | 0.014 | -0.109 | 0.913 |  | -0.011 | 0.015 | -0.753 | 0.452 |  |
| Posterior cingulate | 0.016 | 0.011 | 1.459 | 0.146 |  | -0.007 | 0.013 | -0.545 | 0.586 |  |
| Precentral | -0.004 | 0.017 | -0.240 | 0.811 |  | -0.001 | 0.015 | -0.036 | 0.971 |  |
| Precuneus | 0.008 | 0.016 | 0.518 | 0.605 |  | 0.001 | 0.016 | 0.051 | 0.960 |  |
| Rostral anterior cingulate | -0.002 | 0.011 | -0.224 | 0.823 |  | -0.004 | 0.013 | -0.269 | 0.788 |  |
| Rostral middle frontal | 0.002 | 0.019 | 0.116 | 0.908 |  | 0.012 | 0.016 | 0.736 | 0.463 |  |
| Superior frontal | -0.055 | 0.020 | -2.822 | 0.005 | ✓ | 0.009 | 0.019 | 0.477 | 0.634 |  |
| Superior parietal | -0.008 | 0.020 | -0.387 | 0.699 |  | 0.017 | 0.014 | 1.215 | 0.226 |  |
| Superior temporal | -0.023 | 0.018 | -1.296 | 0.196 |  | 0.007 | 0.019 | 0.353 | 0.724 |  |
| Supramarginal | 0.003 | 0.020 | 0.168 | 0.867 |  | -0.015 | 0.015 | -0.985 | 0.326 |  |
| Frontal pole | 0.000 | 0.011 | 0.031 | 0.975 |  | -0.010 | 0.010 | -0.981 | 0.328 |  |
| Temporal pole | -0.004 | 0.011 | -0.317 | 0.752 |  | 0.009 | 0.011 | 0.844 | 0.400 |  |
| Transverse temporal | 0.006 | 0.012 | 0.516 | 0.607 |  | 0.011 | 0.012 | 0.858 | 0.392 |  |
| Insula | 0.010 | 0.012 | 0.807 | 0.421 |  | -0.029 | 0.015 | -2.014 | 0.045 |  |
| Discrepancy/income inequality | -0.821 | 0.014 | 60.407 | 0.000 |  | -0.822 | 0.014 | 60.656 | 0.000 |  |
| Stimulation/crime | -0.059 | 0.038 | -1.572 | 0.118 |  | -0.061 | 0.038 | -1.589 | 0.114 |  |
| Stimulation/population density | -0.055 | 0.016 | -3.398 | 0.001 |  | -0.056 | 0.016 | -3.424 | 0.001 |  |
| Age | 0.009 | 0.008 | 1.211 | 0.227 |  | 0.006 | 0.007 | 0.769 | 0.443 |  |
| Sex | 0.006 | 0.009 | 0.623 | 0.534 |  | -0.008 | 0.011 | -0.780 | 0.436 |  |

**Supplementary table 7**

*Cortical thickness and area regions predicting discrepancy/neighborhood income inequality objective measure, accounting for stimulation and deprivation objective factors, age and sex as fixed effects, with family and scanner as a random effect, SE=standard error, β=standardized β coefficient, ✓ denotes that result passed Bonferroni correction threshold of p<0.0167.*

|  | Cortical thickness | | | |  | Cortical area | | | |  |
| --- | --- | --- | --- | --- | --- | --- | --- | --- | --- | --- |
|  | β | SE | t-value | p-value | Pass Bonferroni? | β | SE | t-value | p-value | Pass Bonferroni? |
| Intercept | -0.021 | 0.059 | -0.353 | 0.724 |  | -0.017 | 0.057 | -0.293 | 0.769 |  |
| Banks of superior temporal sulcus | -0.020 | 0.015 | -1.349 | 0.179 |  | 0.018 | 0.015 | 1.171 | 0.243 |  |
| Caudal anterior cingulate | -0.009 | 0.013 | -0.741 | 0.459 |  | -0.036 | 0.016 | -2.254 | 0.025 |  |
| Caudal middle frontal | 0.029 | 0.020 | 1.470 | 0.143 |  | -0.013 | 0.016 | -0.777 | 0.438 |  |
| Cuneus | 0.008 | 0.017 | 0.482 | 0.630 |  | 0.001 | 0.018 | 0.044 | 0.965 |  |
| Entorhinal | -0.019 | 0.012 | -1.588 | 0.114 |  | 0.009 | 0.013 | 0.714 | 0.476 |  |
| Fusiform | -0.004 | 0.018 | -0.214 | 0.831 |  | -0.025 | 0.018 | -1.385 | 0.167 |  |
| Inferior parietal | -0.029 | 0.026 | -1.105 | 0.270 |  | 0.004 | 0.018 | 0.219 | 0.827 |  |
| Inferior temporal | -0.037 | 0.018 | -2.034 | 0.043 |  | -0.001 | 0.022 | -0.063 | 0.950 |  |
| Isthmus cingulate | 0.008 | 0.012 | 0.625 | 0.533 |  | 0.000 | 0.017 | 0.024 | 0.981 |  |
| Lateral occipital | -0.044 | 0.021 | -2.070 | 0.040 |  | 0.009 | 0.018 | 0.486 | 0.627 |  |
| Lateral orbitofrontal | -0.003 | 0.017 | -0.178 | 0.859 |  | -0.033 | 0.021 | -1.567 | 0.119 |  |
| Lingual | 0.002 | 0.017 | 0.122 | 0.903 |  | 0.008 | 0.019 | 0.419 | 0.676 |  |
| Medial orbitofrontal | 0.026 | 0.015 | 1.678 | 0.095 |  | 0.055 | 0.018 | 3.103 | 0.002 | ✓ |
| Middle temporal | 0.045 | 0.022 | 2.036 | 0.043 |  | -0.013 | 0.025 | -0.534 | 0.594 |  |
| Parahippocampal | 0.001 | 0.012 | 0.114 | 0.909 |  | -0.012 | 0.014 | -0.833 | 0.406 |  |
| Paracentral | -0.002 | 0.018 | -0.126 | 0.900 |  | 0.006 | 0.015 | 0.384 | 0.702 |  |
| Pars opercularis | -0.002 | 0.017 | -0.109 | 0.913 |  | -0.022 | 0.015 | -1.512 | 0.132 |  |
| Pars orbitalis | 0.008 | 0.014 | 0.567 | 0.572 |  | -0.021 | 0.017 | -1.269 | 0.206 |  |
| Parstriangularis | -0.020 | 0.017 | -1.166 | 0.245 |  | 0.000 | 0.014 | 0.010 | 0.992 |  |
| Pericalcarine | -0.015 | 0.015 | -1.016 | 0.311 |  | 0.025 | 0.019 | 1.282 | 0.201 |  |
| Postcentral | -0.005 | 0.017 | -0.317 | 0.751 |  | 0.009 | 0.018 | 0.506 | 0.614 |  |
| Posterior cingulate | 0.008 | 0.013 | 0.592 | 0.555 |  | -0.004 | 0.016 | -0.258 | 0.797 |  |
| Precentral | 0.004 | 0.021 | 0.172 | 0.864 |  | -0.034 | 0.018 | -1.868 | 0.063 |  |
| Precuneus | -0.007 | 0.019 | -0.367 | 0.714 |  | 0.006 | 0.020 | 0.324 | 0.746 |  |
| Rostral anterior cingulate | 0.007 | 0.013 | 0.554 | 0.580 |  | 0.044 | 0.016 | 2.659 | 0.008 | ✓ |
| Rostral middle frontal | 0.024 | 0.024 | 1.035 | 0.302 |  | 0.009 | 0.020 | 0.433 | 0.666 |  |
| Superior frontal | -0.018 | 0.024 | -0.726 | 0.469 |  | -0.007 | 0.025 | -0.279 | 0.781 |  |
| Superior parietal | 0.036 | 0.025 | 1.451 | 0.148 |  | -0.034 | 0.019 | -1.830 | 0.069 |  |
| Superior temporal | 0.011 | 0.021 | 0.521 | 0.603 |  | 0.002 | 0.024 | 0.102 | 0.919 |  |
| Supramarginal | 0.023 | 0.024 | 0.989 | 0.324 |  | 0.001 | 0.018 | 0.077 | 0.939 |  |
| Frontal pole | -0.005 | 0.013 | -0.408 | 0.684 |  | 0.001 | 0.013 | 0.081 | 0.935 |  |
| Temporal pole | 0.020 | 0.013 | 1.535 | 0.126 |  | 0.012 | 0.013 | 0.876 | 0.382 |  |
| Transverse temporal | -0.004 | 0.014 | -0.270 | 0.787 |  | 0.005 | 0.016 | 0.298 | 0.766 |  |
| Insula | -0.018 | 0.015 | -1.254 | 0.211 |  | 0.028 | 0.018 | 1.550 | 0.123 |  |
| Deprivation/median family income | -0.598 | 0.017 | 36.015 | 0.000 |  | -0.595 | 0.017 | 35.851 | 0.000 |  |
| Stimulation/crime | -0.131 | 0.050 | -2.599 | 0.010 |  | -0.138 | 0.049 | -2.792 | 0.006 |  |
| Stimulation/population density | 0.072 | 0.020 | 3.640 | 0.000 |  | 0.072 | 0.020 | 3.691 | 0.000 |  |
| Age | -0.008 | 0.009 | -0.834 | 0.405 |  | -0.013 | 0.009 | -1.500 | 0.135 |  |
| Sex | 0.018 | 0.012 | 1.556 | 0.121 |  | 0.013 | 0.013 | 0.959 | 0.339 |  |

**Supplementary table 8**

*Cortical thickness and area regions predicting stimulation/neighborhood total crimes objective measure, accounting for discrepancy and deprivation objective factors, age and sex as fixed effects, with family and scanner as a random effect, SE=standard error, β=standardized β coefficient, ✓ denotes that result passed Bonferroni correction threshold of p<0.0167.*

|  | Cortical thickness | | | |  | Cortical area | | | |  |
| --- | --- | --- | --- | --- | --- | --- | --- | --- | --- | --- |
|  | β | SE | t-value | p-value | Pass Bonferroni? | β | SE | t-value | p-value | Pass Bonferroni? |
| Intercept | -0.353 | 0.152 | -2.319 | 0.021 |  | -0.355 | 0.152 | -2.334 | 0.020 |  |
| Banks of superior temporal sulcus | -0.001 | 0.001 | -0.821 | 0.413 |  | 0.001 | 0.001 | 1.084 | 0.280 |  |
| Caudal anterior cingulate | -0.001 | 0.001 | -0.657 | 0.513 |  | -0.001 | 0.001 | -1.100 | 0.273 |  |
| Caudal middle frontal | 0.003 | 0.002 | 1.594 | 0.113 |  | -0.001 | 0.001 | -0.382 | 0.703 |  |
| Cuneus | -0.003 | 0.001 | -1.886 | 0.062 |  | 0.001 | 0.002 | 0.609 | 0.544 |  |
| Entorhinal | 0.001 | 0.001 | 1.047 | 0.297 |  | 0.001 | 0.001 | 0.726 | 0.469 |  |
| Fusiform | 0.001 | 0.002 | 0.369 | 0.713 |  | -0.002 | 0.001 | -1.190 | 0.236 |  |
| Inferior parietal | 0.000 | 0.002 | -0.198 | 0.843 |  | 0.001 | 0.002 | 0.309 | 0.757 |  |
| Inferior temporal | -0.002 | 0.002 | -1.435 | 0.154 |  | -0.002 | 0.002 | -1.070 | 0.287 |  |
| Isthmus cingulate | -0.001 | 0.001 | -1.152 | 0.251 |  | -0.001 | 0.001 | -0.407 | 0.685 |  |
| Lateral occipital | 0.002 | 0.002 | 1.033 | 0.304 |  | -0.002 | 0.002 | -1.248 | 0.214 |  |
| Lateral orbitofrontal | -0.002 | 0.001 | -1.221 | 0.224 |  | 0.000 | 0.002 | -0.021 | 0.983 |  |
| Lingual | 0.002 | 0.001 | 1.431 | 0.155 |  | 0.000 | 0.002 | -0.236 | 0.814 |  |
| Medial orbitofrontal | 0.001 | 0.001 | 1.047 | 0.297 |  | 0.001 | 0.002 | 0.822 | 0.412 |  |
| Middle temporal | 0.001 | 0.002 | 0.444 | 0.658 |  | 0.002 | 0.002 | 1.034 | 0.303 |  |
| Parahippocampal | 0.001 | 0.001 | 0.496 | 0.621 |  | 0.000 | 0.001 | -0.245 | 0.807 |  |
| Paracentral | 0.002 | 0.002 | 1.121 | 0.264 |  | -0.001 | 0.001 | -0.739 | 0.461 |  |
| Pars opercularis | -0.001 | 0.002 | -0.967 | 0.335 |  | 0.000 | 0.001 | -0.106 | 0.916 |  |
| Pars orbitalis | 0.001 | 0.001 | 0.688 | 0.493 |  | -0.001 | 0.001 | -0.879 | 0.381 |  |
| Parstriangularis | -0.001 | 0.001 | -1.038 | 0.301 |  | 0.001 | 0.001 | 0.405 | 0.686 |  |
| Pericalcarine | 0.000 | 0.001 | 0.280 | 0.780 |  | 0.000 | 0.002 | 0.062 | 0.951 |  |
| Postcentral | 0.000 | 0.001 | -0.321 | 0.749 |  | 0.000 | 0.002 | 0.077 | 0.938 |  |
| Posterior cingulate | 0.001 | 0.001 | 1.087 | 0.279 |  | 0.001 | 0.001 | 0.647 | 0.519 |  |
| Precentral | -0.003 | 0.002 | -1.556 | 0.122 |  | 0.001 | 0.002 | 0.469 | 0.640 |  |
| Precuneus | -0.002 | 0.002 | -0.902 | 0.369 |  | 0.001 | 0.002 | 0.442 | 0.659 |  |
| Rostral anterior cingulate | 0.001 | 0.001 | 0.497 | 0.620 |  | 0.001 | 0.001 | 0.602 | 0.548 |  |
| Rostral middle frontal | 0.000 | 0.002 | 0.040 | 0.968 |  | 0.001 | 0.002 | 0.739 | 0.461 |  |
| Superior frontal | -0.001 | 0.002 | -0.422 | 0.674 |  | -0.001 | 0.002 | -0.380 | 0.705 |  |
| Superior parietal | 0.000 | 0.002 | -0.030 | 0.976 |  | 0.001 | 0.002 | 0.912 | 0.363 |  |
| Superior temporal | 0.003 | 0.002 | 1.320 | 0.189 |  | -0.003 | 0.002 | -1.400 | 0.164 |  |
| Supramarginal | 0.002 | 0.002 | 0.992 | 0.323 |  | 0.001 | 0.001 | 0.915 | 0.362 |  |
| Frontal pole | -0.001 | 0.001 | -0.831 | 0.407 |  | 0.000 | 0.001 | 0.091 | 0.928 |  |
| Temporal pole | 0.001 | 0.001 | 0.564 | 0.574 |  | 0.000 | 0.001 | -0.004 | 0.997 |  |
| Transverse temporal | 0.000 | 0.001 | 0.093 | 0.926 |  | 0.000 | 0.001 | -0.015 | 0.988 |  |
| Insula | 0.000 | 0.001 | -0.415 | 0.679 |  | 0.000 | 0.002 | -0.150 | 0.881 |  |
| Deprivation/median family income | 0.002 | 0.006 | 0.363 | 0.717 |  | 0.001 | 0.006 | 0.103 | 0.918 |  |
| Discrepancy/income inequality | 0.002 | 0.006 | 0.328 | 0.743 |  | -0.002 | 0.006 | -0.422 | 0.674 |  |
| Stimulation/population density | 0.002 | 0.006 | 0.388 | 0.699 |  | 0.006 | 0.006 | 1.058 | 0.292 |  |
| Age | 0.000 | 0.001 | -0.196 | 0.845 |  | 0.000 | 0.001 | 0.183 | 0.855 |  |
| Sex | 0.001 | 0.001 | 1.099 | 0.274 |  | 0.001 | 0.001 | 0.462 | 0.645 |  |

**Supplementary table 9**

*Cortical thickness and area regions predicting stimulation/neighborhood population density objective measure, accounting for discrepancy and deprivation objective factors, age and sex as fixed effects, with family and scanner as a random effect, SE=standard error, β=standardized β coefficient, ✓ denotes that result passed Bonferroni correction threshold of p<0.0167.*

|  | Cortical thickness | | | |  | Cortical area | | | |  |
| --- | --- | --- | --- | --- | --- | --- | --- | --- | --- | --- |
|  | β | SE | t-value | p-value | Pass Bonferroni? | β | SE | t-value | p-value | Pass Bonferroni? |
| Intercept | 0.358 | 0.234 | 1.528 | 0.127 |  | 0.365 | 0.235 | 1.557 | 0.120 |  |
| Banks of superior temporal sulcus | -0.003 | 0.010 | -0.285 | 0.776 |  | 0.015 | 0.012 | 1.254 | 0.212 |  |
| Caudal anterior cingulate | 0.021 | 0.009 | 2.421 | 0.016 | ✓ | 0.011 | 0.012 | 0.886 | 0.377 |  |
| Caudal middle frontal | -0.015 | 0.014 | -1.087 | 0.279 |  | 0.012 | 0.012 | 1.007 | 0.315 |  |
| Cuneus | 0.011 | 0.012 | 0.899 | 0.370 |  | -0.016 | 0.014 | -1.155 | 0.249 |  |
| Entorhinal | -0.007 | 0.009 | -0.730 | 0.466 |  | -0.012 | 0.011 | -1.107 | 0.270 |  |
| Fusiform | 0.031 | 0.013 | 2.333 | 0.021 |  | 0.007 | 0.014 | 0.518 | 0.605 |  |
| Inferior parietal | -0.036 | 0.017 | -2.150 | 0.033 |  | -0.011 | 0.014 | -0.780 | 0.437 |  |
| Inferior temporal | -0.025 | 0.014 | -1.858 | 0.065 |  | -0.008 | 0.016 | -0.486 | 0.628 |  |
| Isthmus cingulate | -0.011 | 0.008 | -1.287 | 0.200 |  | -0.005 | 0.012 | -0.461 | 0.646 |  |
| Lateral occipital | 0.051 | 0.016 | 3.125 | 0.002 | ✓ | 0.021 | 0.013 | 1.580 | 0.116 |  |
| Lateral orbitofrontal | 0.024 | 0.011 | 2.211 | 0.028 |  | -0.020 | 0.016 | -1.262 | 0.209 |  |
| Lingual | 0.005 | 0.013 | 0.421 | 0.674 |  | 0.013 | 0.015 | 0.918 | 0.360 |  |
| Medial orbitofrontal | 0.000 | 0.010 | 0.012 | 0.991 |  | 0.004 | 0.014 | 0.318 | 0.751 |  |
| Middle temporal | 0.033 | 0.016 | 2.148 | 0.033 |  | 0.002 | 0.019 | 0.114 | 0.909 |  |
| Parahippocampal | -0.006 | 0.009 | -0.651 | 0.516 |  | 0.002 | 0.012 | 0.159 | 0.874 |  |
| Paracentral | -0.008 | 0.012 | -0.638 | 0.524 |  | -0.031 | 0.012 | -2.614 | 0.010 | ✓ |
| Pars opercularis | -0.021 | 0.012 | -1.703 | 0.090 |  | 0.004 | 0.011 | 0.343 | 0.732 |  |
| Pars orbitalis | -0.006 | 0.010 | -0.593 | 0.554 |  | 0.014 | 0.013 | 1.122 | 0.263 |  |
| Parstriangularis | 0.029 | 0.012 | 2.384 | 0.018 |  | -0.006 | 0.011 | -0.520 | 0.604 |  |
| Pericalcarine | -0.025 | 0.011 | -2.321 | 0.021 |  | -0.018 | 0.015 | -1.224 | 0.223 |  |
| Postcentral | 0.016 | 0.012 | 1.335 | 0.184 |  | 0.008 | 0.014 | 0.573 | 0.567 |  |
| Posterior cingulate | -0.017 | 0.009 | -1.801 | 0.073 |  | -0.018 | 0.011 | -1.571 | 0.118 |  |
| Precentral | -0.055 | 0.014 | -3.850 | 0.000 | ✓ | -0.004 | 0.014 | -0.257 | 0.798 |  |
| Precuneus | 0.040 | 0.013 | 3.023 | 0.003 | ✓ | -0.007 | 0.014 | -0.460 | 0.646 |  |
| Rostral anterior cingulate | -0.005 | 0.009 | -0.516 | 0.606 |  | -0.008 | 0.013 | -0.662 | 0.509 |  |
| Rostral middle frontal | -0.008 | 0.016 | -0.496 | 0.620 |  | -0.034 | 0.015 | -2.229 | 0.027 |  |
| Superior frontal | 0.031 | 0.017 | 1.852 | 0.066 |  | 0.035 | 0.018 | 1.929 | 0.055 |  |
| Superior parietal | -0.023 | 0.016 | -1.399 | 0.164 |  | 0.000 | 0.013 | 0.001 | 1.000 |  |
| Superior temporal | 0.003 | 0.014 | 0.187 | 0.852 |  | -0.031 | 0.018 | -1.659 | 0.099 |  |
| Supramarginal | 0.008 | 0.017 | 0.465 | 0.643 |  | 0.024 | 0.013 | 1.821 | 0.070 |  |
| Frontal pole | -0.014 | 0.009 | -1.611 | 0.109 |  | 0.001 | 0.010 | 0.052 | 0.958 |  |
| Temporal pole | -0.003 | 0.009 | -0.386 | 0.700 |  | 0.011 | 0.010 | 1.039 | 0.300 |  |
| Transverse temporal | -0.014 | 0.010 | -1.507 | 0.134 |  | 0.003 | 0.012 | 0.256 | 0.798 |  |
| Insula | -0.025 | 0.010 | -2.502 | 0.013 | ✓ | 0.023 | 0.014 | 1.660 | 0.099 |  |
| Deprivation/median family income | 0.066 | 0.023 | 2.873 | 0.005 |  | 0.061 | 0.023 | 2.638 | 0.009 |  |
| Discrepancy/income inequality | 0.177 | 0.022 | 8.095 | 0.000 |  | 0.174 | 0.022 | 7.860 | 0.000 |  |
| Stimulation/crime | 0.784 | 0.088 | 8.878 | 0.000 |  | 0.800 | 0.088 | 9.037 | 0.000 |  |
| Age | -0.002 | 0.006 | -0.241 | 0.810 |  | -0.001 | 0.006 | -0.136 | 0.892 |  |
| Sex | -0.008 | 0.008 | -1.072 | 0.285 |  | -0.007 | 0.010 | -0.727 | 0.468 |  |

**Supplementary table 10**

*Subcortical volume regions predicting domains, accounting for age and sex and other domains as fixed effects, with family and scanner as a random effect, SE=standard error, β=standardized β coefficient, ✓ denotes that result passed Bonferroni correction threshold of p<0.0167.*

| **Discrepancy/sense of belonging** | β | SE | t-value | p-value | Pass Bonferroni? |
| --- | --- | --- | --- | --- | --- |
| Intercept | -0.014 | 0.029 | -0.494 | 0.622 |  |
| Thalamus | 0.021 | 0.027 | 0.801 | 0.424 |  |
| Caudate | 0.005 | 0.023 | 0.222 | 0.824 |  |
| Putamen | 0.007 | 0.024 | 0.297 | 0.767 |  |
| Pallidum | -0.018 | 0.024 | -0.753 | 0.452 |  |
| Hippocampus | -0.014 | 0.025 | -0.549 | 0.584 |  |
| Amygdala | 0.003 | 0.024 | 0.117 | 0.907 |  |
| Accumbens | 0.018 | 0.023 | 0.803 | 0.423 |  |
| Stimulation factor | -0.015 | 0.021 | -0.746 | 0.456 |  |
| Deprivation factor | 0.003 | 0.023 | 0.132 | 0.895 |  |
| Discrepancy/American culture participation | 0.149 | 0.022 | 6.711 | 0.000 |  |
| Age | 0.033 | 0.015 | 2.189 | 0.030 |  |
| Sex | 0.007 | 0.020 | 0.345 | 0.730 |  |
| **Stimulation/neighborhood safety** | β | SE | t-value | p-value | Pass Bonferroni? |
| Intercept | 0.050 | 0.044 | 1.137 | 0.256 |  |
| Thalamus | 0.020 | 0.031 | 0.659 | 0.511 |  |
| Caudate | 0.049 | 0.027 | 1.830 | 0.069 |  |
| Putamen | -0.005 | 0.028 | -0.170 | 0.865 |  |
| Pallidum | -0.008 | 0.030 | -0.285 | 0.776 |  |
| Hippocampus | 0.029 | 0.031 | 0.925 | 0.357 |  |
| Amygdala | 0.026 | 0.031 | 0.851 | 0.396 |  |
| Accumbens | -0.065 | 0.028 | -2.352 | 0.020 |  |
| Discrepancy/American culture participation | 0.049 | 0.023 | 2.157 | 0.033 |  |
| Deprivation/deprivation | -0.142 | 0.022 | -6.421 | 0.000 |  |
| Discrepancy/belonging with ethnic group | -0.023 | 0.022 | -1.046 | 0.297 |  |
| Age | -0.023 | 0.020 | -1.154 | 0.250 |  |
| Sex | 0.011 | 0.023 | 0.479 | 0.633 |  |
| **Deprivation self-report** | β | SE | t-value | p-value | Pass Bonferroni? |
| Intercept | -0.012 | 0.044 | -0.267 | 0.790 |  |
| Thalamus | -0.028 | 0.034 | -0.818 | 0.415 |  |
| Caudate | -0.041 | 0.028 | -1.491 | 0.138 |  |
| Putamen | -0.011 | 0.028 | -0.395 | 0.694 |  |
| Pallidum | -0.006 | 0.029 | -0.210 | 0.834 |  |
| Hippocampus | 0.006 | 0.031 | 0.190 | 0.849 |  |
| Amygdala | 0.015 | 0.030 | 0.486 | 0.628 |  |
| Accumbens | -0.021 | 0.028 | -0.776 | 0.439 |  |
| Stimulation/neighborhood safety | -0.104 | 0.023 | -4.566 | 0.000 |  |
| Discrepancy/American culture participation | -0.054 | 0.027 | -1.996 | 0.048 |  |
| Discrepancy/Belonging with ethnic group | 0.027 | 0.026 | 1.054 | 0.294 |  |
| Age | 0.038 | 0.018 | 2.113 | 0.036 |  |
| Sex | 0.050 | 0.024 | 2.122 | 0.036 |  |
| **Discrepancy/American culture participation** | β | SE | t-value | p-value | Pass Bonferroni? |
| Intercept | 0.015 | 0.038 | 0.386 | 0.700 |  |
| Thalamus | 0.032 | 0.025 | 1.285 | 0.200 |  |
| Caudate | -0.012 | 0.020 | -0.576 | 0.566 |  |
| Putamen | 0.005 | 0.022 | 0.221 | 0.825 |  |
| Pallidum | -0.005 | 0.022 | -0.243 | 0.808 |  |
| Hippocampus | 0.004 | 0.024 | 0.170 | 0.866 |  |
| Amygdala | -0.010 | 0.023 | -0.414 | 0.679 |  |
| Accumbens | -0.004 | 0.021 | -0.206 | 0.837 |  |
| Stimulation/neighborhood safety | 0.054 | 0.019 | 2.822 | 0.005 |  |
| Deprivation/deprivation | -0.072 | 0.023 | -3.204 | 0.002 |  |
| Discrepancy/belonging | 0.160 | 0.021 | 7.575 | 0.000 |  |
| Age | -0.004 | 0.013 | -0.302 | 0.763 |  |
| Sex | 0.007 | 0.019 | 0.371 | 0.711 |  |
| **Median family income** | β | SE | t-value | p-value | Pass Bonferroni? |
| Intercept | 0.032 | 0.037 | 0.876 | 0.381 |  |
| Thalamus | 0.012 | 0.013 | 0.915 | 0.361 |  |
| Caudate | 0.022 | 0.012 | 1.878 | 0.062 |  |
| Putamen | -0.019 | 0.012 | -1.568 | 0.118 |  |
| Pallidum | 0.018 | 0.012 | 1.468 | 0.144 |  |
| Hippocampus | 0.023 | 0.014 | 1.594 | 0.112 |  |
| Amygdala | 0.013 | 0.013 | 0.959 | 0.339 |  |
| Accumbens | -0.023 | 0.012 | -1.903 | 0.058 |  |
| Age | 0.005 | 0.007 | 0.667 | 0.505 |  |
| Sex | -0.006 | 0.010 | -0.600 | 0.549 |  |
| Discrepancy/income inequality | -0.824 | 0.013 | -61.479 | 0.000 |  |
| Stimulation/crime | -0.061 | 0.037 | -1.671 | 0.096 |  |
| Stimulation/population density | -0.054 | 0.016 | -3.393 | 0.001 |  |
| **Discrepancy/income inequality** | β | SE | t-value | p-value | Pass Bonferroni? |
| Intercept | -0.020 | 0.058 | -0.343 | 0.732 |  |
| Thalamus | 0.021 | 0.016 | 1.321 | 0.188 |  |
| Caudate | -0.030 | 0.014 | -2.098 | 0.037 |  |
| Putamen | 0.008 | 0.015 | 0.535 | 0.593 |  |
| Pallidum | -0.009 | 0.014 | -0.587 | 0.558 |  |
| Hippocampus | -0.039 | 0.018 | -2.230 | 0.027 |  |
| Amygdala | 0.017 | 0.016 | 1.058 | 0.291 |  |
| Accumbens | 0.016 | 0.015 | 1.045 | 0.297 |  |
| Age | -0.007 | 0.009 | -0.805 | 0.422 |  |
| Sex | 0.014 | 0.012 | 1.236 | 0.218 |  |
| Deprivation/median family income | -0.602 | 0.017 | -36.415 | 0.000 |  |
| Stimulation/crime | -0.131 | 0.050 | -2.626 | 0.009 |  |
| Stimulation/population density | 0.069 | 0.020 | 3.508 | 0.001 |  |
| **Stimulation/crime** | β | SE | t-value | p-value | Pass Bonferroni? |
| Intercept | -0.355 | 0.152 | -2.328 | 0.020 |  |
| Thalamus | 0.000 | 0.001 | 0.219 | 0.827 |  |
| Caudate | 0.000 | 0.001 | 0.046 | 0.963 |  |
| Putamen | 0.000 | 0.001 | 0.243 | 0.808 |  |
| Pallidum | 0.000 | 0.001 | -0.312 | 0.755 |  |
| Hippocampus | 0.001 | 0.001 | 0.396 | 0.693 |  |
| Amygdala | -0.002 | 0.001 | -1.276 | 0.204 |  |
| Accumbens | 0.000 | 0.001 | 0.318 | 0.751 |  |
| Deprivation/median family income | 0.003 | 0.006 | 0.413 | 0.680 |  |
| Discrepancy/income inequality | -0.001 | 0.005 | -0.180 | 0.857 |  |
| Stimulation/population density | 0.004 | 0.006 | 0.777 | 0.438 |  |
| Age | 0.000 | 0.001 | 0.115 | 0.909 |  |
| Sex | 0.000 | 0.001 | 0.141 | 0.888 |  |
| **Stimulation/population density** | β | SE | t-value | p-value | Pass Bonferroni? |
| Intercept | 0.363 | 0.235 | 1.549 | 0.122 |  |
| Thalamus | 0.010 | 0.013 | 0.775 | 0.439 |  |
| Caudate | -0.018 | 0.011 | -1.597 | 0.112 |  |
| Putamen | 0.011 | 0.012 | 0.956 | 0.340 |  |
| Pallidum | 0.012 | 0.011 | 1.039 | 0.300 |  |
| Hippocampus | -0.021 | 0.014 | -1.523 | 0.129 |  |
| Amygdala | 0.004 | 0.013 | 0.335 | 0.738 |  |
| Accumbens | -0.009 | 0.011 | -0.792 | 0.429 |  |
| Deprivation/median family income | 0.063 | 0.023 | 2.722 | 0.007 |  |
| Discrepancy/income inequality | 0.174 | 0.022 | 7.849 | 0.000 |  |
| Stimulation/crime | 0.801 | 0.088 | 9.063 | 0.000 |  |
| Age | -0.005 | 0.006 | -0.823 | 0.412 |  |
| Sex | -0.003 | 0.009 | -0.329 | 0.742 |  |

**Supplementary table 11**

*Cortical thickness and area regions predicting discrepancy/sense of belonging with ethnic group self-report factor, accounting for stimulation and deprivation self-report factors, age and sex as fixed effects, with family and scanner as a random effect, SE=standard error, β=standardized β coefficient (analyses in the whole sample), ✓ denotes that result passed Bonferroni correction threshold of p<0.0167.*

|  | Cortical thickness | | | |  | Cortical area | | | |  |
| --- | --- | --- | --- | --- | --- | --- | --- | --- | --- | --- |
|  | β | SE | t-value | p-value | Pass Bonferroni? | β | SE | t-value | p-value | Pass Bonferroni? |
| Intercept | -0.009 | 0.022 | -0.394 | 0.694 |  | -0.001 | 0.021 | -0.068 | 0.946 |  |
| Banks of superior temporal sulcus | -0.001 | 0.014 | -0.095 | 0.924 |  | 0.004 | 0.014 | 0.275 | 0.784 |  |
| Caudal anterior cingulate | 0.001 | 0.012 | 0.090 | 0.929 |  | -0.005 | 0.015 | -0.359 | 0.720 |  |
| Caudal middle frontal | -0.024 | 0.018 | -1.374 | 0.170 |  | -0.020 | 0.015 | -1.370 | 0.171 |  |
| Cuneus | -0.008 | 0.015 | -0.528 | 0.598 |  | 0.004 | 0.017 | 0.257 | 0.797 |  |
| Entorhinal | 0.014 | 0.011 | 1.217 | 0.224 |  | 0.005 | 0.012 | 0.413 | 0.680 |  |
| Fusiform | -0.005 | 0.017 | -0.327 | 0.744 |  | 0.017 | 0.017 | 0.998 | 0.319 |  |
| Inferior parietal | -0.011 | 0.023 | -0.491 | 0.624 |  | 0.003 | 0.017 | 0.192 | 0.848 |  |
| Inferior temporal | 0.002 | 0.016 | 0.128 | 0.898 |  | -0.038 | 0.019 | -1.960 | 0.050 |  |
| Isthmus cingulate | -0.002 | 0.011 | -0.165 | 0.869 |  | 0.038 | 0.015 | 2.528 | 0.012 | ✓ |
| Lateral occipital | -0.070 | 0.020 | -3.546 | 0.000 | ✓ | -0.002 | 0.016 | -0.132 | 0.895 |  |
| Lateral orbitofrontal | -0.024 | 0.015 | -1.618 | 0.106 |  | -0.020 | 0.019 | -1.077 | 0.282 |  |
| Lingual | -0.022 | 0.015 | -1.440 | 0.150 |  | -0.030 | 0.017 | -1.743 | 0.082 |  |
| Medial orbitofrontal | 0.020 | 0.014 | 1.405 | 0.160 |  | 0.015 | 0.017 | 0.881 | 0.379 |  |
| Middle temporal | -0.024 | 0.019 | -1.281 | 0.201 |  | 0.000 | 0.022 | -0.009 | 0.993 |  |
| Parahippocampal | -0.006 | 0.011 | -0.575 | 0.566 |  | -0.005 | 0.012 | -0.438 | 0.661 |  |
| Paracentral | -0.025 | 0.017 | -1.480 | 0.139 |  | 0.010 | 0.014 | 0.757 | 0.449 |  |
| Pars opercularis | 0.013 | 0.015 | 0.889 | 0.374 |  | 0.000 | 0.014 | -0.016 | 0.987 |  |
| Pars orbitalis | -0.003 | 0.013 | -0.226 | 0.822 |  | -0.027 | 0.016 | -1.663 | 0.097 |  |
| Parstriangularis | 0.017 | 0.016 | 1.085 | 0.278 |  | 0.000 | 0.014 | 0.021 | 0.984 |  |
| Pericalcarine | -0.012 | 0.014 | -0.909 | 0.364 |  | 0.033 | 0.018 | 1.800 | 0.072 |  |
| Postcentral | -0.017 | 0.016 | -1.062 | 0.289 |  | -0.001 | 0.017 | -0.079 | 0.937 |  |
| Posterior cingulate | 0.016 | 0.012 | 1.274 | 0.203 |  | 0.007 | 0.016 | 0.429 | 0.668 |  |
| Precentral | 0.026 | 0.019 | 1.407 | 0.160 |  | 0.002 | 0.017 | 0.104 | 0.917 |  |
| Precuneus | 0.022 | 0.018 | 1.224 | 0.221 |  | -0.036 | 0.018 | -2.064 | 0.039 |  |
| Rostral anterior cingulate | 0.011 | 0.012 | 0.919 | 0.359 |  | 0.021 | 0.016 | 1.324 | 0.186 |  |
| Rostral middle frontal | 0.007 | 0.022 | 0.311 | 0.756 |  | 0.003 | 0.018 | 0.135 | 0.892 |  |
| Superior frontal | 0.013 | 0.022 | 0.607 | 0.544 |  | -0.006 | 0.023 | -0.270 | 0.787 |  |
| Superior parietal | 0.019 | 0.022 | 0.873 | 0.383 |  | -0.031 | 0.016 | -1.882 | 0.060 |  |
| Superior temporal | 0.020 | 0.018 | 1.079 | 0.281 |  | 0.015 | 0.022 | 0.682 | 0.496 |  |
| Supramarginal | -0.001 | 0.022 | -0.032 | 0.975 |  | 0.023 | 0.017 | 1.356 | 0.175 |  |
| Frontal pole | -0.010 | 0.012 | -0.839 | 0.402 |  | -0.034 | 0.012 | -2.815 | 0.005 | ✓ |
| Temporal pole | -0.001 | 0.012 | -0.120 | 0.904 |  | -0.015 | 0.012 | -1.199 | 0.231 |  |
| Transverse temporal | 0.000 | 0.013 | -0.019 | 0.985 |  | 0.014 | 0.014 | 0.991 | 0.322 |  |
| Insula | -0.019 | 0.013 | -1.449 | 0.148 |  | 0.000 | 0.017 | 0.000 | 1.000 |  |
| Stimulation factor | -0.026 | 0.012 | -2.216 | 0.027 |  | -0.029 | 0.012 | -2.478 | 0.013 |  |
| Deprivation factor | 0.045 | 0.013 | 3.551 | 0.000 |  | 0.043 | 0.013 | 3.367 | 0.001 |  |
| Discrepancy/American culture participation | 0.229 | 0.012 | 18.583 | 0.000 |  | 0.229 | 0.012 | 18.525 | 0.000 |  |
| Age | 0.006 | 0.009 | 0.599 | 0.549 |  | 0.016 | 0.009 | 1.776 | 0.076 |  |
| Sex | 0.012 | 0.011 | 1.093 | 0.275 |  | 0.026 | 0.012 | 2.079 | 0.038 |  |

**Supplementary table 12**

*Cortical thickness and area regions predicting stimulation/neighborhood safety self-report factor, accounting for discrepancy and deprivation self-report factors, age and sex as fixed effects, with family and scanner as a random effect, SE=standard error, β=standardized β coefficient (analyses in the whole sample), ✓ denotes that result passed Bonferroni correction threshold of p<0.0167.*

|  | Cortical thickness | | | |  | Cortical area | | | |  |
| --- | --- | --- | --- | --- | --- | --- | --- | --- | --- | --- |
|  | β | SE | t-value | p-value | Pass Bonferroni? | β | SE | t-value | p-value | Pass Bonferroni? |
| Intercept | 0.015 | 0.051 | 0.297 | 0.766 |  | 0.007 | 0.052 | 0.134 | 0.894 |  |
| Banks of superior temporal sulcus | 0.036 | 0.015 | 2.387 | 0.017 |  | -0.025 | 0.016 | -1.617 | 0.106 |  |
| Caudal anterior cingulate | -0.025 | 0.013 | -1.940 | 0.053 |  | -0.001 | 0.017 | -0.067 | 0.946 |  |
| Caudal middle frontal | -0.002 | 0.020 | -0.112 | 0.911 |  | 0.007 | 0.016 | 0.397 | 0.692 |  |
| Cuneus | -0.010 | 0.017 | -0.586 | 0.558 |  | -0.022 | 0.019 | -1.152 | 0.250 |  |
| Entorhinal | -0.016 | 0.012 | -1.315 | 0.189 |  | 0.031 | 0.013 | 2.337 | 0.020 |  |
| Fusiform | -0.011 | 0.018 | -0.579 | 0.563 |  | -0.007 | 0.019 | -0.345 | 0.730 |  |
| Inferior parietal | -0.081 | 0.025 | -3.206 | 0.001 | ✓ | -0.027 | 0.019 | -1.470 | 0.142 |  |
| Inferior temporal | -0.010 | 0.018 | -0.536 | 0.592 |  | -0.004 | 0.021 | -0.193 | 0.847 |  |
| Isthmus cingulate | -0.016 | 0.012 | -1.308 | 0.191 |  | -0.037 | 0.016 | -2.258 | 0.024 |  |
| Lateral occipital | 0.085 | 0.022 | 3.845 | 0.000 | ✓ | 0.049 | 0.018 | 2.687 | 0.007 | ✓ |
| Lateral orbitofrontal | 0.042 | 0.017 | 2.463 | 0.014 | ✓ | -0.007 | 0.021 | -0.309 | 0.757 |  |
| Lingual | 0.043 | 0.017 | 2.516 | 0.012 | ✓ | 0.008 | 0.019 | 0.433 | 0.665 |  |
| Medial orbitofrontal | -0.018 | 0.016 | -1.139 | 0.255 |  | -0.001 | 0.019 | -0.069 | 0.945 |  |
| Middle temporal | 0.029 | 0.021 | 1.412 | 0.159 |  | 0.063 | 0.024 | 2.616 | 0.009 | ✓ |
| Parahippocampal | 0.015 | 0.012 | 1.227 | 0.220 |  | 0.016 | 0.014 | 1.123 | 0.262 |  |
| Paracentral | 0.005 | 0.018 | 0.296 | 0.767 |  | -0.033 | 0.015 | -2.158 | 0.031 |  |
| Pars opercularis | -0.014 | 0.017 | -0.838 | 0.402 |  | 0.011 | 0.015 | 0.722 | 0.470 |  |
| Pars orbitalis | -0.023 | 0.015 | -1.567 | 0.118 |  | 0.050 | 0.018 | 2.825 | 0.005 | ✓ |
| Parstriangularis | -0.044 | 0.018 | -2.470 | 0.014 | ✓ | -0.015 | 0.015 | -0.995 | 0.320 |  |
| Pericalcarine | -0.013 | 0.015 | -0.867 | 0.386 |  | -0.006 | 0.020 | -0.284 | 0.777 |  |
| Postcentral | 0.021 | 0.017 | 1.241 | 0.215 |  | -0.004 | 0.019 | -0.211 | 0.833 |  |
| Posterior cingulate | 0.008 | 0.013 | 0.563 | 0.573 |  | 0.018 | 0.017 | 1.054 | 0.292 |  |
| Precentral | 0.013 | 0.020 | 0.627 | 0.531 |  | 0.022 | 0.019 | 1.165 | 0.244 |  |
| Precuneus | 0.010 | 0.020 | 0.488 | 0.626 |  | 0.009 | 0.019 | 0.477 | 0.633 |  |
| Rostral anterior cingulate | 0.010 | 0.014 | 0.754 | 0.451 |  | 0.016 | 0.018 | 0.903 | 0.367 |  |
| Rostral middle frontal | 0.048 | 0.024 | 1.956 | 0.051 |  | -0.016 | 0.021 | -0.757 | 0.449 |  |
| Superior frontal | -0.044 | 0.024 | -1.849 | 0.065 |  | 0.002 | 0.025 | 0.081 | 0.935 |  |
| Superior parietal | 0.015 | 0.025 | 0.619 | 0.536 |  | 0.004 | 0.018 | 0.240 | 0.810 |  |
| Superior temporal | -0.021 | 0.020 | -1.060 | 0.289 |  | 0.020 | 0.025 | 0.800 | 0.424 |  |
| Supramarginal | 0.017 | 0.024 | 0.681 | 0.496 |  | 0.021 | 0.019 | 1.114 | 0.266 |  |
| Frontal pole | -0.019 | 0.013 | -1.457 | 0.146 |  | 0.015 | 0.014 | 1.076 | 0.282 |  |
| Temporal pole | 0.012 | 0.013 | 0.930 | 0.353 |  | -0.014 | 0.014 | -0.991 | 0.322 |  |
| Transverse temporal | 0.008 | 0.015 | 0.555 | 0.579 |  | -0.036 | 0.016 | -2.268 | 0.024 |  |
| Insula | -0.005 | 0.014 | -0.317 | 0.751 |  | -0.008 | 0.019 | -0.445 | 0.656 |  |
| Discrepancy/American culture participation | 0.127 | 0.012 | 10.447 | 0.000 |  | 0.126 | 0.012 | 10.382 | 0.000 |  |
| Deprivation/deprivation | -0.191 | 0.012 | 15.855 | 0.000 |  | -0.188 | 0.012 | 15.600 | 0.000 |  |
| Discrepancy/Sense of belonging with ethnic group | -0.024 | 0.012 | -2.042 | 0.042 |  | -0.027 | 0.012 | -2.259 | 0.024 |  |
| Age | 0.005 | 0.011 | 0.455 | 0.649 |  | -0.003 | 0.011 | -0.268 | 0.789 |  |
| Sex | -0.001 | 0.012 | -0.117 | 0.907 |  | -0.028 | 0.013 | -2.099 | 0.036 |  |

**Supplementary table 13**

*Cortical thickness and area regions predicting deprivation/deprivation self-report factor, accounting for discrepancy and deprivation self-report factors, age and sex as fixed effects, with family and scanner as a random effect, SE=standard error, β=standardized β coefficient (analyses in the whole sample), ✓ denotes that result passed Bonferroni correction threshold of p<0.0167.*

|  | Cortical thickness | | | |  | Cortical area | | | |  |
| --- | --- | --- | --- | --- | --- | --- | --- | --- | --- | --- |
|  | β | SE | t-value | p-value | Pass Bonferroni? | β | SE | t-value | p-value | Pass Bonferroni? |
| Intercept | -0.006 | 0.041 | -0.148 | 0.883 |  | 0.007 | 0.040 | 0.178 | 0.859 |  |
| Banks of superior temporal sulcus | -0.013 | 0.012 | -1.104 | 0.270 |  | 0.003 | 0.012 | 0.264 | 0.792 |  |
| Caudal anterior cingulate | -0.015 | 0.010 | -1.501 | 0.134 |  | -0.023 | 0.013 | -1.777 | 0.076 |  |
| Caudal middle frontal | 0.030 | 0.016 | 1.930 | 0.054 |  | -0.015 | 0.013 | -1.124 | 0.261 |  |
| Cuneus | -0.016 | 0.013 | -1.181 | 0.238 |  | 0.011 | 0.015 | 0.714 | 0.475 |  |
| Entorhinal | 0.003 | 0.010 | 0.332 | 0.740 |  | 0.003 | 0.010 | 0.322 | 0.748 |  |
| Fusiform | -0.003 | 0.015 | -0.230 | 0.818 |  | 0.019 | 0.015 | 1.259 | 0.209 |  |
| Inferior parietal | 0.033 | 0.020 | 1.662 | 0.097 |  | -0.003 | 0.015 | -0.233 | 0.816 |  |
| Inferior temporal | 0.000 | 0.014 | -0.014 | 0.989 |  | -0.054 | 0.017 | -3.220 | 0.001 | ✓ |
| Isthmus cingulate | -0.003 | 0.010 | -0.327 | 0.743 |  | 0.012 | 0.013 | 0.941 | 0.347 |  |
| Lateral occipital | -0.055 | 0.018 | -3.078 | 0.002 | ✓ | -0.024 | 0.015 | -1.676 | 0.094 |  |
| Lateral orbitofrontal | -0.015 | 0.013 | -1.147 | 0.252 |  | 0.000 | 0.017 | 0.003 | 0.998 |  |
| Lingual | -0.001 | 0.013 | -0.082 | 0.935 |  | -0.018 | 0.015 | -1.153 | 0.249 |  |
| Medial orbitofrontal | 0.019 | 0.012 | 1.569 | 0.117 |  | 0.004 | 0.015 | 0.255 | 0.799 |  |
| Middle temporal | -0.019 | 0.017 | -1.142 | 0.254 |  | 0.016 | 0.019 | 0.841 | 0.400 |  |
| Parahippocampal | -0.021 | 0.010 | -2.180 | 0.030 |  | 0.013 | 0.011 | 1.210 | 0.227 |  |
| Paracentral | 0.008 | 0.015 | 0.554 | 0.580 |  | -0.010 | 0.012 | -0.827 | 0.409 |  |
| Pars opercularis | 0.011 | 0.013 | 0.808 | 0.419 |  | -0.005 | 0.012 | -0.458 | 0.647 |  |
| Pars orbitalis | 0.006 | 0.011 | 0.501 | 0.616 |  | -0.028 | 0.014 | -2.007 | 0.045 |  |
| Parstriangularis | 0.008 | 0.014 | 0.575 | 0.565 |  | -0.004 | 0.012 | -0.325 | 0.745 |  |
| Pericalcarine | 0.008 | 0.012 | 0.673 | 0.501 |  | 0.021 | 0.016 | 1.287 | 0.199 |  |
| Postcentral | -0.022 | 0.014 | -1.561 | 0.119 |  | -0.009 | 0.015 | -0.585 | 0.559 |  |
| Posterior cingulate | 0.013 | 0.011 | 1.180 | 0.239 |  | -0.003 | 0.014 | -0.222 | 0.825 |  |
| Precentral | -0.057 | 0.016 | -3.452 | 0.001 | ✓ | -0.037 | 0.015 | -2.493 | 0.013 | ✓ |
| Precuneus | 0.003 | 0.016 | 0.181 | 0.857 |  | -0.002 | 0.015 | -0.157 | 0.875 |  |
| Rostral anterior cingulate | 0.027 | 0.011 | 2.582 | 0.010 | ✓ | 0.019 | 0.014 | 1.419 | 0.156 |  |
| Rostral middle frontal | -0.004 | 0.020 | -0.206 | 0.837 |  | 0.011 | 0.016 | 0.710 | 0.478 |  |
| Superior frontal | 0.016 | 0.019 | 0.817 | 0.414 |  | -0.021 | 0.020 | -1.056 | 0.291 |  |
| Superior parietal | 0.024 | 0.020 | 1.242 | 0.215 |  | -0.035 | 0.014 | -2.404 | 0.016 | ✓ |
| Superior temporal | 0.000 | 0.016 | -0.016 | 0.988 |  | -0.001 | 0.020 | -0.052 | 0.959 |  |
| Supramarginal | 0.015 | 0.020 | 0.752 | 0.452 |  | 0.005 | 0.015 | 0.311 | 0.756 |  |
| Frontal pole | 0.001 | 0.010 | 0.115 | 0.909 |  | -0.008 | 0.010 | -0.741 | 0.459 |  |
| Temporal pole | -0.007 | 0.010 | -0.656 | 0.512 |  | 0.003 | 0.011 | 0.278 | 0.781 |  |
| Transverse temporal | 0.009 | 0.011 | 0.823 | 0.411 |  | 0.004 | 0.012 | 0.334 | 0.739 |  |
| Insula | -0.031 | 0.012 | -2.637 | 0.009 | ✓ | 0.030 | 0.015 | 2.034 | 0.042 |  |
| Stimulation/neighborhood safety | -0.148 | 0.011 | 13.903 | 0.000 |  | -0.144 | 0.011 | 13.684 | 0.000 |  |
| Discrepancy/American culture participation | -0.070 | 0.012 | -5.772 | 0.000 |  | -0.067 | 0.012 | -5.545 | 0.000 |  |
| Discrepancy/Sense of belonging with ethnic group | 0.036 | 0.012 | 3.134 | 0.002 |  | 0.034 | 0.012 | 2.919 | 0.004 |  |
| Age | 0.004 | 0.008 | 0.490 | 0.624 |  | 0.006 | 0.008 | 0.831 | 0.406 |  |
| Sex | 0.023 | 0.010 | 2.324 | 0.020 |  | 0.056 | 0.011 | 5.050 | 0.000 |  |

**Supplementary table 14**

*Cortical thickness and area regions predicting discrepancy/American culture participation self-report factor, accounting for stimulation and deprivation self-report factors, age and sex as fixed effects, with family and scanner as a random effect, SE=standard error, β=standardized β coefficient (analyses in the whole sample), ✓ denotes that result passed Bonferroni correction threshold of p<0.0167.*

|  | Cortical thickness | | | |  | Cortical area | | | |  |
| --- | --- | --- | --- | --- | --- | --- | --- | --- | --- | --- |
|  | β | SE | t-value | p-value | Pass Bonferroni? | β | SE | t-value | p-value | Pass Bonferroni? |
| Intercept | -0.016 | 0.022 | -0.715 | 0.475 |  | -0.017 | 0.022 | -0.774 | 0.439 |  |
| Banks of superior temporal sulcus | -0.002 | 0.012 | -0.130 | 0.896 |  | -0.001 | 0.012 | -0.108 | 0.914 |  |
| Caudal anterior cingulate | -0.003 | 0.010 | -0.281 | 0.779 |  | 0.006 | 0.013 | 0.452 | 0.651 |  |
| Caudal middle frontal | -0.018 | 0.016 | -1.134 | 0.257 |  | 0.023 | 0.013 | 1.775 | 0.076 |  |
| Cuneus | 0.011 | 0.013 | 0.844 | 0.399 |  | 0.018 | 0.015 | 1.186 | 0.236 |  |
| Entorhinal | -0.009 | 0.010 | -0.927 | 0.354 |  | 0.011 | 0.011 | 0.998 | 0.319 |  |
| Fusiform | -0.019 | 0.015 | -1.323 | 0.186 |  | 0.012 | 0.015 | 0.796 | 0.426 |  |
| Inferior parietal | 0.011 | 0.020 | 0.568 | 0.570 |  | -0.007 | 0.015 | -0.456 | 0.649 |  |
| Inferior temporal | 0.017 | 0.014 | 1.194 | 0.233 |  | 0.007 | 0.017 | 0.410 | 0.682 |  |
| Isthmus cingulate | -0.005 | 0.010 | -0.543 | 0.587 |  | -0.025 | 0.013 | -1.902 | 0.058 |  |
| Lateral occipital | -0.004 | 0.018 | -0.248 | 0.804 |  | 0.003 | 0.015 | 0.224 | 0.823 |  |
| Lateral orbitofrontal | 0.010 | 0.013 | 0.721 | 0.471 |  | -0.008 | 0.017 | -0.453 | 0.651 |  |
| Lingual | 0.019 | 0.013 | 1.386 | 0.166 |  | -0.021 | 0.015 | -1.385 | 0.167 |  |
| Medial orbitofrontal | -0.023 | 0.012 | -1.876 | 0.061 |  | -0.005 | 0.015 | -0.323 | 0.747 |  |
| Middle temporal | 0.005 | 0.017 | 0.327 | 0.744 |  | 0.007 | 0.019 | 0.365 | 0.716 |  |
| Parahippocampal | -0.005 | 0.010 | -0.564 | 0.573 |  | -0.008 | 0.011 | -0.735 | 0.463 |  |
| Paracentral | 0.016 | 0.015 | 1.062 | 0.289 |  | -0.018 | 0.012 | -1.494 | 0.136 |  |
| Pars opercularis | -0.046 | 0.013 | -3.442 | 0.001 | ✓ | 0.016 | 0.012 | 1.383 | 0.167 |  |
| Pars orbitalis | -0.013 | 0.011 | -1.115 | 0.265 |  | 0.031 | 0.014 | 2.195 | 0.028 |  |
| Parstriangularis | -0.003 | 0.014 | -0.238 | 0.812 |  | -0.009 | 0.012 | -0.765 | 0.445 |  |
| Pericalcarine | -0.021 | 0.012 | -1.708 | 0.088 |  | 0.020 | 0.016 | 1.232 | 0.218 |  |
| Postcentral | -0.006 | 0.014 | -0.411 | 0.681 |  | -0.023 | 0.015 | -1.511 | 0.131 |  |
| Posterior cingulate | -0.020 | 0.011 | -1.860 | 0.063 |  | -0.003 | 0.014 | -0.185 | 0.853 |  |
| Precentral | -0.030 | 0.017 | -1.805 | 0.071 |  | 0.021 | 0.015 | 1.428 | 0.154 |  |
| Precuneus | 0.009 | 0.016 | 0.556 | 0.578 |  | 0.011 | 0.016 | 0.730 | 0.465 |  |
| Rostral anterior cingulate | 0.001 | 0.011 | 0.113 | 0.910 |  | -0.025 | 0.014 | -1.770 | 0.077 |  |
| Rostral middle frontal | 0.036 | 0.019 | 1.861 | 0.063 |  | 0.002 | 0.016 | 0.106 | 0.916 |  |
| Superior frontal | 0.019 | 0.019 | 1.008 | 0.314 |  | 0.008 | 0.020 | 0.388 | 0.698 |  |
| Superior parietal | -0.001 | 0.020 | -0.060 | 0.952 |  | 0.000 | 0.015 | 0.031 | 0.976 |  |
| Superior temporal | 0.011 | 0.016 | 0.654 | 0.514 |  | -0.011 | 0.020 | -0.580 | 0.562 |  |
| Supramarginal | 0.007 | 0.019 | 0.355 | 0.722 |  | 0.004 | 0.015 | 0.244 | 0.807 |  |
| Frontal pole | 0.002 | 0.010 | 0.188 | 0.851 |  | 0.011 | 0.011 | 1.042 | 0.298 |  |
| Temporal pole | 0.010 | 0.010 | 0.938 | 0.349 |  | -0.008 | 0.011 | -0.739 | 0.460 |  |
| Transverse temporal | 0.019 | 0.011 | 1.670 | 0.095 |  | -0.017 | 0.012 | -1.331 | 0.184 |  |
| Insula | 0.027 | 0.012 | 2.298 | 0.022 |  | 0.008 | 0.015 | 0.559 | 0.576 |  |
| Stimulation/neighborhood safety | 0.103 | 0.011 | 9.642 | 0.000 |  | 0.103 | 0.011 | 9.628 | 0.000 |  |
| Deprivation/deprivation | -0.072 | 0.012 | -5.978 | 0.000 |  | -0.071 | 0.012 | -5.811 | 0.000 |  |
| Discrepancy/belonging | 0.202 | 0.011 | 17.803 | 0.000 |  | 0.202 | 0.011 | 17.813 | 0.000 |  |
| Age | 0.019 | 0.008 | 2.332 | 0.020 |  | 0.011 | 0.008 | 1.417 | 0.157 |  |
| Sex | -0.004 | 0.010 | -0.398 | 0.691 |  | -0.008 | 0.011 | -0.706 | 0.480 |  |

**Supplementary table 15**

*Cortical thickness and area regions predicting deprivation/neighborhood median family income objective measure, accounting for stimulation and discrepancy objective factors, age and sex as fixed effects, with family and scanner as a random effect, SE=standard error, β=standardized β coefficient (analyses in the whole sample), ✓ denotes that result passed Bonferroni correction threshold of p<0.0167.*

|  | Cortical thickness | | | |  | Cortical area | | | |  |
| --- | --- | --- | --- | --- | --- | --- | --- | --- | --- | --- |
|  | β | SE | t-value | p-value | Pass Bonferroni? | β | SE | t-value | p-value | Pass Bonferroni? |
| Intercept | 0.105 | 0.084 | 1.247 | 0.213 |  | 0.104 | 0.084 | 1.241 | 0.215 |  |
| Banks of superior temporal sulcus | 0.001 | 0.002 | 0.462 | 0.644 |  | 0.000 | 0.003 | -0.046 | 0.963 |  |
| Caudal anterior cingulate | 0.001 | 0.002 | 0.228 | 0.820 |  | 0.000 | 0.003 | -0.027 | 0.979 |  |
| Caudal middle frontal | 0.001 | 0.003 | 0.395 | 0.693 |  | 0.003 | 0.003 | 1.079 | 0.281 |  |
| Cuneus | 0.000 | 0.003 | -0.062 | 0.951 |  | 0.002 | 0.003 | 0.571 | 0.568 |  |
| Entorhinal | -0.001 | 0.002 | -0.652 | 0.514 |  | 0.003 | 0.002 | 1.136 | 0.256 |  |
| Fusiform | 0.000 | 0.003 | -0.099 | 0.921 |  | -0.005 | 0.003 | -1.575 | 0.116 |  |
| Inferior parietal | 0.000 | 0.005 | -0.072 | 0.942 |  | 0.002 | 0.003 | 0.559 | 0.576 |  |
| Inferior temporal | 0.001 | 0.003 | 0.361 | 0.718 |  | 0.004 | 0.004 | 1.202 | 0.230 |  |
| Isthmus cingulate | 0.000 | 0.002 | -0.189 | 0.850 |  | -0.002 | 0.003 | -0.711 | 0.478 |  |
| Lateral occipital | 0.000 | 0.004 | 0.066 | 0.948 |  | 0.003 | 0.003 | 1.032 | 0.303 |  |
| Lateral orbitofrontal | 0.001 | 0.003 | 0.397 | 0.691 |  | -0.001 | 0.004 | -0.142 | 0.887 |  |
| Lingual | 0.001 | 0.003 | 0.438 | 0.662 |  | -0.001 | 0.003 | -0.341 | 0.733 |  |
| Medial orbitofrontal | 0.000 | 0.003 | -0.018 | 0.986 |  | 0.001 | 0.003 | 0.379 | 0.704 |  |
| Middle temporal | -0.008 | 0.004 | -2.154 | 0.031 |  | -0.001 | 0.004 | -0.161 | 0.872 |  |
| Parahippocampal | 0.003 | 0.002 | 1.523 | 0.128 |  | 0.001 | 0.002 | 0.533 | 0.594 |  |
| Paracentral | 0.003 | 0.003 | 0.909 | 0.364 |  | -0.001 | 0.003 | -0.384 | 0.701 |  |
| Pars opercularis | 0.001 | 0.003 | 0.428 | 0.669 |  | -0.002 | 0.003 | -0.935 | 0.350 |  |
| Pars orbitalis | -0.002 | 0.002 | -0.735 | 0.463 |  | 0.000 | 0.003 | -0.155 | 0.877 |  |
| Parstriangularis | -0.003 | 0.003 | -0.912 | 0.362 |  | 0.002 | 0.003 | 0.615 | 0.539 |  |
| Pericalcarine | -0.002 | 0.003 | -0.567 | 0.571 |  | -0.001 | 0.004 | -0.340 | 0.734 |  |
| Postcentral | 0.005 | 0.003 | 1.625 | 0.104 |  | 0.003 | 0.003 | 1.062 | 0.289 |  |
| Posterior cingulate | 0.000 | 0.002 | -0.032 | 0.975 |  | -0.001 | 0.003 | -0.194 | 0.846 |  |
| Precentral | 0.002 | 0.004 | 0.419 | 0.675 |  | 0.002 | 0.003 | 0.504 | 0.614 |  |
| Precuneus | 0.002 | 0.004 | 0.502 | 0.616 |  | 0.001 | 0.004 | 0.417 | 0.676 |  |
| Rostral anterior cingulate | -0.002 | 0.002 | -1.066 | 0.287 |  | 0.003 | 0.003 | 1.143 | 0.253 |  |
| Rostral middle frontal | 0.002 | 0.004 | 0.420 | 0.675 |  | -0.001 | 0.004 | -0.387 | 0.699 |  |
| Superior frontal | 0.000 | 0.004 | -0.092 | 0.927 |  | 0.000 | 0.004 | 0.011 | 0.992 |  |
| Superior parietal | -0.005 | 0.004 | -1.061 | 0.289 |  | 0.000 | 0.003 | -0.063 | 0.950 |  |
| Superior temporal | 0.003 | 0.004 | 0.749 | 0.454 |  | 0.006 | 0.004 | 1.467 | 0.143 |  |
| Supramarginal | -0.002 | 0.004 | -0.494 | 0.621 |  | 0.002 | 0.003 | 0.680 | 0.497 |  |
| Frontal pole | -0.001 | 0.002 | -0.232 | 0.817 |  | 0.003 | 0.002 | 1.172 | 0.241 |  |
| Temporal pole | 0.000 | 0.002 | -0.140 | 0.889 |  | -0.002 | 0.002 | -0.774 | 0.439 |  |
| Transverse temporal | 0.001 | 0.002 | 0.588 | 0.557 |  | -0.001 | 0.003 | -0.470 | 0.639 |  |
| Insula | 0.005 | 0.003 | 1.787 | 0.074 |  | -0.005 | 0.003 | -1.472 | 0.141 |  |
| Discrepancy/income inequality | -0.620 | 0.007 | 94.302 | 0.000 |  | -0.620 | 0.007 | 94.094 | 0.000 |  |
| Stimulation/crime | 0.114 | 0.017 | 6.682 | 0.000 |  | 0.111 | 0.017 | 6.535 | 0.000 |  |
| Stimulation/population density | -0.077 | 0.008 | -9.666 | 0.000 |  | -0.075 | 0.008 | -9.500 | 0.000 |  |
| Age | 0.000 | 0.002 | 0.040 | 0.968 |  | -0.001 | 0.002 | -0.592 | 0.554 |  |
| Sex | 0.005 | 0.002 | 2.051 | 0.041 |  | -0.001 | 0.003 | -0.285 | 0.776 |  |

**Supplementary table 16**

*Cortical thickness and area regions predicting discrepancy/neighborhood income inequality objective measure, accounting for stimulation and deprivation objective factors, age and sex as fixed effects, with family and scanner as a random effect, SE=standard error, β=standardized β coefficient (analyses in the whole sample), ✓ denotes that result passed Bonferroni correction threshold of p<0.0167.*

|  | Cortical thickness | | | |  | Cortical area | | | |  |
| --- | --- | --- | --- | --- | --- | --- | --- | --- | --- | --- |
|  | β | SE | t-value | p-value | Pass Bonferroni? | β | SE | t-value | p-value | Pass Bonferroni? |
| Intercept | 0.026 | 0.042 | 0.609 | 0.542 |  | 0.027 | 0.042 | 0.653 | 0.514 |  |
| Banks of superior temporal sulcus | -0.002 | 0.004 | -0.558 | 0.577 |  | 0.007 | 0.004 | 1.849 | 0.065 |  |
| Caudal anterior cingulate | 0.000 | 0.003 | 0.141 | 0.888 |  | -0.007 | 0.004 | -1.657 | 0.098 |  |
| Caudal middle frontal | 0.008 | 0.005 | 1.454 | 0.146 |  | 0.005 | 0.004 | 1.166 | 0.244 |  |
| Cuneus | 0.002 | 0.004 | 0.502 | 0.616 |  | -0.003 | 0.005 | -0.570 | 0.569 |  |
| Entorhinal | -0.001 | 0.003 | -0.429 | 0.668 |  | 0.003 | 0.003 | 0.944 | 0.345 |  |
| Fusiform | -0.004 | 0.005 | -0.820 | 0.412 |  | -0.009 | 0.005 | -1.915 | 0.056 |  |
| Inferior parietal | 0.007 | 0.007 | 1.071 | 0.284 |  | 0.004 | 0.005 | 0.762 | 0.447 |  |
| Inferior temporal | 0.005 | 0.005 | 1.157 | 0.248 |  | 0.000 | 0.006 | -0.073 | 0.942 |  |
| Isthmus cingulate | -0.002 | 0.003 | -0.573 | 0.567 |  | 0.004 | 0.004 | 0.980 | 0.327 |  |
| Lateral occipital | -0.013 | 0.006 | -2.182 | 0.029 |  | 0.001 | 0.005 | 0.124 | 0.901 |  |
| Lateral orbitofrontal | -0.006 | 0.004 | -1.383 | 0.167 |  | -0.002 | 0.006 | -0.354 | 0.724 |  |
| Lingual | 0.000 | 0.004 | 0.095 | 0.924 |  | -0.004 | 0.005 | -0.701 | 0.483 |  |
| Medial orbitofrontal | 0.007 | 0.004 | 1.796 | 0.073 |  | 0.003 | 0.005 | 0.658 | 0.511 |  |
| Middle temporal | -0.015 | 0.006 | -2.638 | 0.008 | ✓ | -0.008 | 0.006 | -1.324 | 0.186 |  |
| Parahippocampal | -0.002 | 0.003 | -0.693 | 0.488 |  | 0.001 | 0.004 | 0.230 | 0.818 |  |
| Paracentral | 0.000 | 0.005 | -0.091 | 0.928 |  | -0.002 | 0.004 | -0.469 | 0.639 |  |
| Pars opercularis | 0.008 | 0.004 | 1.909 | 0.057 |  | -0.005 | 0.004 | -1.355 | 0.176 |  |
| Pars orbitalis | -0.001 | 0.004 | -0.136 | 0.892 |  | -0.010 | 0.005 | -2.139 | 0.033 |  |
| Parstriangularis | -0.003 | 0.004 | -0.739 | 0.460 |  | 0.006 | 0.004 | 1.478 | 0.140 |  |
| Pericalcarine | -0.003 | 0.004 | -0.718 | 0.473 |  | 0.003 | 0.006 | 0.616 | 0.538 |  |
| Postcentral | -0.001 | 0.005 | -0.162 | 0.871 |  | 0.002 | 0.005 | 0.343 | 0.732 |  |
| Posterior cingulate | 0.002 | 0.004 | 0.467 | 0.641 |  | 0.006 | 0.004 | 1.369 | 0.171 |  |
| Precentral | -0.002 | 0.006 | -0.303 | 0.762 |  | -0.004 | 0.005 | -0.766 | 0.444 |  |
| Precuneus | -0.007 | 0.005 | -1.269 | 0.205 |  | -0.006 | 0.005 | -1.133 | 0.257 |  |
| Rostral anterior cingulate | -0.001 | 0.004 | -0.332 | 0.740 |  | 0.008 | 0.004 | 1.842 | 0.066 |  |
| Rostral middle frontal | -0.002 | 0.006 | -0.315 | 0.753 |  | 0.005 | 0.005 | 0.850 | 0.395 |  |
| Superior frontal | 0.006 | 0.006 | 0.865 | 0.387 |  | 0.001 | 0.007 | 0.085 | 0.932 |  |
| Superior parietal | 0.000 | 0.007 | -0.002 | 0.999 |  | -0.008 | 0.005 | -1.755 | 0.079 |  |
| Superior temporal | 0.007 | 0.005 | 1.262 | 0.207 |  | -0.004 | 0.006 | -0.569 | 0.569 |  |
| Supramarginal | -0.002 | 0.007 | -0.269 | 0.788 |  | 0.004 | 0.005 | 0.797 | 0.425 |  |
| Frontal pole | -0.001 | 0.003 | -0.394 | 0.694 |  | 0.004 | 0.003 | 1.102 | 0.271 |  |
| Temporal pole | 0.000 | 0.003 | -0.077 | 0.938 |  | 0.002 | 0.004 | 0.617 | 0.537 |  |
| Transverse temporal | 0.001 | 0.004 | 0.245 | 0.806 |  | 0.002 | 0.004 | 0.383 | 0.702 |  |
| Insula | -0.001 | 0.004 | -0.247 | 0.805 |  | 0.003 | 0.005 | 0.587 | 0.557 |  |
| Deprivation/median family income | -0.704 | 0.008 | 90.442 | 0.000 |  | -0.705 | 0.008 | 90.371 | 0.000 |  |
| Stimulation/crime | 0.058 | 0.019 | 2.991 | 0.003 |  | 0.059 | 0.019 | 3.087 | 0.002 |  |
| Stimulation/population density | 0.113 | 0.008 | 13.564 | 0.000 |  | 0.112 | 0.008 | 13.461 | 0.000 |  |
| Age | -0.001 | 0.003 | -0.483 | 0.629 |  | 0.000 | 0.002 | 0.161 | 0.872 |  |
| Sex | 0.005 | 0.003 | 1.389 | 0.165 |  | 0.005 | 0.004 | 1.165 | 0.244 |  |

**Supplementary table 17**

*Cortical thickness and area regions predicting stimulation/neighborhood total crimes objective measure, accounting for discrepancy and deprivation objective factors, age and sex as fixed effects, with family and scanner as a random effect, SE=standard error, β=standardized β coefficient (analyses in the whole sample), ✓ denotes that result passed Bonferroni correction threshold of p<0.0167.*

|  | Cortical thickness | | | |  | Cortical area | | | |  |
| --- | --- | --- | --- | --- | --- | --- | --- | --- | --- | --- |
|  | β | SE | t-value | p-value | Pass Bonferroni? | β | SE | t-value | p-value | Pass Bonferroni? |
| Intercept | 0.011 | 0.167 | 0.068 | 0.946 |  | 0.012 | 0.167 | 0.072 | 0.943 |  |
| Banks of superior temporal sulcus | 0.001 | 0.002 | 0.450 | 0.653 |  | 0.000 | 0.003 | 0.131 | 0.896 |  |
| Caudal anterior cingulate | 0.003 | 0.002 | 1.530 | 0.126 |  | 0.000 | 0.003 | -0.023 | 0.982 |  |
| Caudal middle frontal | -0.002 | 0.003 | -0.682 | 0.495 |  | -0.002 | 0.003 | -0.750 | 0.453 |  |
| Cuneus | -0.002 | 0.003 | -0.689 | 0.491 |  | 0.002 | 0.003 | 0.498 | 0.619 |  |
| Entorhinal | 0.001 | 0.002 | 0.318 | 0.750 |  | 0.001 | 0.002 | 0.370 | 0.712 |  |
| Fusiform | -0.002 | 0.003 | -0.587 | 0.557 |  | -0.006 | 0.003 | -2.012 | 0.044 |  |
| Inferior parietal | -0.008 | 0.004 | -1.809 | 0.071 |  | -0.003 | 0.003 | -0.880 | 0.379 |  |
| Inferior temporal | 0.000 | 0.003 | -0.019 | 0.985 |  | 0.005 | 0.004 | 1.394 | 0.164 |  |
| Isthmus cingulate | 0.006 | 0.002 | 3.042 | 0.002 | ✓ | 0.002 | 0.003 | 0.623 | 0.534 |  |
| Lateral occipital | 0.002 | 0.004 | 0.639 | 0.523 |  | -0.003 | 0.003 | -1.115 | 0.265 |  |
| Lateral orbitofrontal | -0.003 | 0.003 | -0.973 | 0.331 |  | -0.003 | 0.004 | -0.722 | 0.470 |  |
| Lingual | -0.003 | 0.003 | -1.093 | 0.274 |  | 0.003 | 0.003 | 1.036 | 0.300 |  |
| Medial orbitofrontal | -0.004 | 0.003 | -1.663 | 0.097 |  | 0.007 | 0.003 | 2.275 | 0.023 |  |
| Middle temporal | -0.010 | 0.004 | -2.763 | 0.006 | ✓ | -0.003 | 0.004 | -0.814 | 0.416 |  |
| Parahippocampal | -0.002 | 0.002 | -0.834 | 0.405 |  | 0.002 | 0.002 | 1.001 | 0.317 |  |
| Paracentral | -0.004 | 0.003 | -1.413 | 0.158 |  | 0.002 | 0.003 | 0.747 | 0.455 |  |
| Pars opercularis | 0.001 | 0.003 | 0.456 | 0.649 |  | -0.004 | 0.002 | -1.522 | 0.128 |  |
| Pars orbitalis | 0.005 | 0.002 | 1.964 | 0.050 |  | 0.002 | 0.003 | 0.526 | 0.599 |  |
| Parstriangularis | -0.005 | 0.003 | -1.593 | 0.112 |  | -0.001 | 0.002 | -0.475 | 0.635 |  |
| Pericalcarine | 0.000 | 0.003 | -0.090 | 0.928 |  | 0.000 | 0.004 | -0.086 | 0.931 |  |
| Postcentral | -0.002 | 0.003 | -0.611 | 0.541 |  | 0.002 | 0.003 | 0.482 | 0.630 |  |
| Posterior cingulate | -0.004 | 0.002 | -1.671 | 0.095 |  | 0.000 | 0.003 | 0.164 | 0.870 |  |
| Precentral | 0.001 | 0.004 | 0.200 | 0.842 |  | -0.001 | 0.003 | -0.158 | 0.875 |  |
| Precuneus | 0.002 | 0.003 | 0.715 | 0.475 |  | -0.003 | 0.003 | -1.002 | 0.317 |  |
| Rostral anterior cingulate | 0.003 | 0.002 | 1.441 | 0.150 |  | -0.002 | 0.003 | -0.658 | 0.511 |  |
| Rostral middle frontal | 0.008 | 0.004 | 1.992 | 0.047 |  | 0.002 | 0.003 | 0.456 | 0.649 |  |
| Superior frontal | 0.002 | 0.004 | 0.440 | 0.660 |  | 0.004 | 0.004 | 0.885 | 0.377 |  |
| Superior parietal | 0.007 | 0.004 | 1.704 | 0.089 |  | 0.002 | 0.003 | 0.647 | 0.518 |  |
| Superior temporal | 0.003 | 0.003 | 0.899 | 0.369 |  | 0.003 | 0.004 | 0.832 | 0.406 |  |
| Supramarginal | -0.003 | 0.004 | -0.691 | 0.490 |  | -0.004 | 0.003 | -1.234 | 0.217 |  |
| Frontal pole | 0.000 | 0.002 | -0.045 | 0.965 |  | -0.005 | 0.002 | -2.323 | 0.020 |  |
| Temporal pole | -0.001 | 0.002 | -0.300 | 0.765 |  | 0.001 | 0.002 | 0.615 | 0.539 |  |
| Transverse temporal | 0.001 | 0.002 | 0.559 | 0.576 |  | 0.000 | 0.003 | 0.147 | 0.883 |  |
| Insula | -0.001 | 0.002 | -0.581 | 0.561 |  | 0.000 | 0.003 | 0.008 | 0.994 |  |
| Deprivation/median family income | 0.018 | 0.004 | 4.050 | 0.000 |  | 0.017 | 0.004 | 3.876 | 0.000 |  |
| Discrepancy/income inequality | 0.022 | 0.004 | 5.240 | 0.000 |  | 0.023 | 0.004 | 5.342 | 0.000 |  |
| Stimulation/population density | 0.085 | 0.004 | 23.493 | 0.000 |  | 0.085 | 0.004 | 23.475 | 0.000 |  |
| Age | -0.001 | 0.002 | -0.396 | 0.692 |  | 0.000 | 0.002 | -0.211 | 0.833 |  |
| Sex | 0.001 | 0.002 | 0.470 | 0.639 |  | 0.001 | 0.002 | 0.523 | 0.601 |  |

**Supplementary table 18**

*Cortical thickness and area regions predicting stimulation/neighborhood population density objective measure, accounting for discrepancy and deprivation objective factors, age and sex as fixed effects, with family and scanner as a random effect, SE=standard error, β=standardized β coefficient (analyses in the whole sample), ✓ denotes that result passed Bonferroni correction threshold of p<0.0167.*

|  | Cortical thickness | | | |  | Cortical area | | | |  |
| --- | --- | --- | --- | --- | --- | --- | --- | --- | --- | --- |
|  | β | SE | t-value | p-value | Pass Bonferroni? | β | SE | t-value | p-value | Pass Bonferroni? |
| Intercept | 0.288 | 0.288 | 1.001 | 0.317 |  | 0.286 | 0.287 | 0.997 | 0.319 |  |
| Banks of superior temporal sulcus | -0.001 | 0.004 | -0.223 | 0.824 |  | -0.004 | 0.004 | -1.009 | 0.313 |  |
| Caudal anterior cingulate | 0.005 | 0.003 | 1.405 | 0.160 |  | 0.005 | 0.004 | 1.211 | 0.226 |  |
| Caudal middle frontal | -0.002 | 0.005 | -0.419 | 0.676 |  | 0.001 | 0.004 | 0.212 | 0.832 |  |
| Cuneus | 0.002 | 0.004 | 0.540 | 0.589 |  | -0.004 | 0.005 | -0.687 | 0.492 |  |
| Entorhinal | 0.003 | 0.003 | 0.930 | 0.352 |  | -0.005 | 0.003 | -1.331 | 0.183 |  |
| Fusiform | 0.001 | 0.005 | 0.211 | 0.833 |  | 0.008 | 0.005 | 1.533 | 0.126 |  |
| Inferior parietal | -0.002 | 0.007 | -0.333 | 0.739 |  | -0.005 | 0.005 | -0.989 | 0.323 |  |
| Inferior temporal | -0.008 | 0.005 | -1.591 | 0.112 |  | 0.003 | 0.006 | 0.441 | 0.659 |  |
| Isthmus cingulate | -0.006 | 0.003 | -1.854 | 0.064 |  | -0.006 | 0.005 | -1.236 | 0.217 |  |
| Lateral occipital | 0.017 | 0.006 | 2.681 | 0.007 | ✓ | 0.007 | 0.005 | 1.364 | 0.173 |  |
| Lateral orbitofrontal | 0.005 | 0.004 | 1.155 | 0.248 |  | -0.001 | 0.006 | -0.218 | 0.828 |  |
| Lingual | 0.003 | 0.005 | 0.659 | 0.510 |  | -0.001 | 0.005 | -0.219 | 0.826 |  |
| Medial orbitofrontal | 0.001 | 0.004 | 0.210 | 0.834 |  | -0.003 | 0.005 | -0.516 | 0.606 |  |
| Middle temporal | 0.010 | 0.006 | 1.616 | 0.106 |  | -0.001 | 0.007 | -0.174 | 0.862 |  |
| Parahippocampal | 0.002 | 0.003 | 0.471 | 0.638 |  | -0.003 | 0.004 | -0.772 | 0.440 |  |
| Paracentral | -0.001 | 0.005 | -0.255 | 0.799 |  | -0.004 | 0.004 | -0.886 | 0.376 |  |
| Pars opercularis | -0.010 | 0.005 | -2.250 | 0.025 |  | 0.003 | 0.004 | 0.855 | 0.393 |  |
| Pars orbitalis | -0.003 | 0.004 | -0.766 | 0.444 |  | 0.007 | 0.005 | 1.504 | 0.133 |  |
| Parstriangularis | 0.008 | 0.005 | 1.664 | 0.096 |  | -0.003 | 0.004 | -0.856 | 0.392 |  |
| Pericalcarine | -0.011 | 0.004 | -2.706 | 0.007 | ✓ | -0.001 | 0.006 | -0.150 | 0.881 |  |
| Postcentral | 0.004 | 0.005 | 0.912 | 0.362 |  | 0.005 | 0.005 | 1.008 | 0.314 |  |
| Posterior cingulate | -0.004 | 0.004 | -0.999 | 0.318 |  | -0.006 | 0.004 | -1.283 | 0.200 |  |
| Precentral | -0.012 | 0.006 | -2.020 | 0.044 |  | -0.003 | 0.005 | -0.605 | 0.545 |  |
| Precuneus | 0.015 | 0.005 | 2.715 | 0.007 | ✓ | 0.001 | 0.005 | 0.104 | 0.917 |  |
| Rostral anterior cingulate | -0.003 | 0.004 | -0.932 | 0.351 |  | -0.010 | 0.005 | -2.245 | 0.025 |  |
| Rostral middle frontal | -0.007 | 0.007 | -1.047 | 0.295 |  | -0.009 | 0.006 | -1.544 | 0.123 |  |
| Superior frontal | 0.011 | 0.007 | 1.650 | 0.099 |  | 0.012 | 0.007 | 1.751 | 0.080 |  |
| Superior parietal | -0.007 | 0.007 | -0.977 | 0.329 |  | -0.005 | 0.005 | -0.934 | 0.350 |  |
| Superior temporal | -0.001 | 0.006 | -0.153 | 0.878 |  | -0.003 | 0.007 | -0.410 | 0.682 |  |
| Supramarginal | 0.002 | 0.007 | 0.299 | 0.765 |  | 0.000 | 0.005 | -0.026 | 0.979 |  |
| Frontal pole | -0.004 | 0.003 | -1.123 | 0.262 |  | 0.001 | 0.004 | 0.305 | 0.760 |  |
| Temporal pole | -0.002 | 0.003 | -0.562 | 0.574 |  | -0.001 | 0.004 | -0.354 | 0.723 |  |
| Transverse temporal | -0.002 | 0.004 | -0.555 | 0.579 |  | -0.002 | 0.004 | -0.592 | 0.554 |  |
| Insula | -0.004 | 0.004 | -1.053 | 0.292 |  | 0.007 | 0.005 | 1.348 | 0.178 |  |
| Deprivation/median family income | -0.108 | 0.012 | -9.031 | 0.000 |  | -0.106 | 0.012 | -8.868 | 0.000 |  |
| Discrepancy/income inequality | 0.135 | 0.011 | 12.111 | 0.000 |  | 0.135 | 0.011 | 12.087 | 0.000 |  |
| Stimulation/crime | 0.545 | 0.023 | 23.964 | 0.000 |  | 0.546 | 0.023 | 24.047 | 0.000 |  |
| Age | 0.002 | 0.003 | 0.629 | 0.530 |  | 0.001 | 0.003 | 0.569 | 0.569 |  |
| Sex | -0.002 | 0.003 | -0.567 | 0.571 |  | 0.002 | 0.004 | 0.487 | 0.627 |  |

**Supplementary table 19**

*Subcortical volume regions predicting domains, accounting for age and sex as fixed effects, with family and scanner as a random effect, SE=standard error, β=standardized β coefficient (analyses in the whole sample), ✓ denotes that result passed Bonferroni correction threshold of p<0.0167.*

| **Discrepancy/sense of belonging** | β | SE | t-value | p-value | Pass Bonferroni? |
| --- | --- | --- | --- | --- | --- |
| Intercept | -0.008 | 0.023 | -0.341 | 0.733 |  |
| Thalamus | -0.018 | 0.016 | -1.117 | 0.264 |  |
| Caudate | -0.001 | 0.014 | -0.040 | 0.968 |  |
| Putamen | -0.005 | 0.014 | -0.361 | 0.718 |  |
| Pallidum | -0.013 | 0.014 | -0.899 | 0.369 |  |
| Hippocampus | 0.005 | 0.015 | 0.299 | 0.765 |  |
| Amygdala | -0.025 | 0.015 | -1.657 | 0.098 |  |
| Accumbens | 0.011 | 0.013 | 0.781 | 0.435 |  |
| Stimulation factor | -0.005 | 0.012 | -0.445 | 0.656 |  |
| Deprivation factor | 0.032 | 0.013 | 2.492 | 0.013 |  |
| Age | 0.024 | 0.009 | 2.620 | 0.009 |  |
| Sex | 0.023 | 0.012 | 1.971 | 0.049 |  |
| **Stimulation/neighborhood safety** | β | SE | t-value | p-value |  |
| Intercept | 0.018 | 0.052 | 0.353 | 0.724 |  |
| Thalamus | 0.043 | 0.017 | 2.612 | 0.009 | ✓ |
| Caudate | 0.014 | 0.014 | 1.008 | 0.314 |  |
| Putamen | 0.020 | 0.015 | 1.331 | 0.183 |  |
| Pallidum | -0.030 | 0.015 | -1.935 | 0.053 |  |
| Hippocampus | 0.025 | 0.016 | 1.516 | 0.130 |  |
| Amygdala | 0.044 | 0.017 | 2.647 | 0.008 | ✓ |
| Accumbens | -0.013 | 0.015 | -0.868 | 0.386 |  |
| Discrepancy/American culture participation | 0.130 | 0.012 | 10.767 | 0.000 |  |
| Deprivation/deprivation | -0.192 | 0.012 | -16.003 | 0.000 |  |
| Discrepancy/belonging with ethnic group | -0.031 | 0.012 | -2.567 | 0.010 |  |
| Age | -0.010 | 0.011 | -0.992 | 0.322 |  |
| Sex | -0.020 | 0.012 | -1.632 | 0.103 |  |
| **Deprivation self-report** | β | SE | t-value | p-value |  |
| Intercept | -0.004 | 0.040 | -0.099 | 0.921 |  |
| Thalamus | -0.005 | 0.014 | -0.391 | 0.696 |  |
| Caudate | -0.036 | 0.012 | -3.033 | 0.003 | ✓ |
| Putamen | -0.014 | 0.012 | -1.177 | 0.239 |  |
| Pallidum | 0.002 | 0.012 | 0.170 | 0.865 |  |
| Hippocampus | -0.049 | 0.013 | -3.670 | 0.000 | ✓ |
| Amygdala | 0.000 | 0.013 | -0.037 | 0.971 |  |
| Accumbens | -0.013 | 0.012 | -1.072 | 0.284 |  |
| Stimulation/neighborhood safety | -0.147 | 0.011 | -13.949 | 0.000 |  |
| Discrepancy/American culture participation | -0.071 | 0.012 | -5.869 | 0.000 |  |
| Discrepancy/Belonging with ethnic group | 0.040 | 0.011 | 3.445 | 0.001 |  |
| Age | 0.010 | 0.008 | 1.363 | 0.173 |  |
| Sex | 0.044 | 0.010 | 4.264 | 0.000 |  |
| **Discrepancy/American culture participation** | β | SE | t-value | p-value |  |
| Intercept | -0.015 | 0.022 | -0.681 | 0.496 |  |
| Thalamus | 0.008 | 0.014 | 0.569 | 0.570 |  |
| Caudate | 0.000 | 0.012 | -0.034 | 0.973 |  |
| Putamen | 0.008 | 0.012 | 0.660 | 0.509 |  |
| Pallidum | 0.001 | 0.013 | 0.055 | 0.956 |  |
| Hippocampus | -0.003 | 0.013 | -0.210 | 0.834 |  |
| Amygdala | 0.013 | 0.013 | 0.970 | 0.332 |  |
| Accumbens | -0.013 | 0.012 | -1.123 | 0.262 |  |
| Stimulation/neighborhood safety | 0.104 | 0.011 | 9.804 | 0.000 |  |
| Deprivation/deprivation | -0.074 | 0.012 | -6.117 | 0.000 |  |
| Discrepancy/belonging | 0.200 | 0.011 | 17.746 | 0.000 |  |
| Age | 0.011 | 0.008 | 1.388 | 0.166 |  |
| Sex | -0.004 | 0.010 | -0.380 | 0.704 |  |
| **Median family income** | β | SE | t-value | p-value |  |
| Intercept | 0.106 | 0.085 | 1.253 | 0.210 |  |
| Thalamus | 0.003 | 0.003 | 1.010 | 0.313 |  |
| Caudate | 0.003 | 0.003 | 0.940 | 0.348 |  |
| Putamen | 0.004 | 0.003 | 1.462 | 0.144 |  |
| Pallidum | -0.003 | 0.003 | -1.175 | 0.240 |  |
| Hippocampus | 0.001 | 0.003 | 0.390 | 0.697 |  |
| Amygdala | 0.007 | 0.003 | 2.404 | 0.016 | ✓ |
| Accumbens | -0.003 | 0.003 | -1.084 | 0.279 |  |
| Age | -0.002 | 0.002 | -1.120 | 0.263 |  |
| Sex | 0.001 | 0.002 | 0.412 | 0.680 |  |
| Discrepancy/income inequality | -0.620 | 0.007 | -94.646 | 0.000 |  |
| Stimulation/crime | 0.116 | 0.017 | 6.860 | 0.000 |  |
| Stimulation/population density | -0.077 | 0.008 | -9.788 | 0.000 |  |
| **Discrepancy/income inequality** | β | SE | t-value | p-value |  |
| Intercept | 0.028 | 0.042 | 0.663 | 0.508 |  |
| Thalamus | 0.003 | 0.005 | 0.717 | 0.474 |  |
| Caudate | -0.004 | 0.004 | -1.053 | 0.292 |  |
| Putamen | 0.001 | 0.004 | 0.240 | 0.811 |  |
| Pallidum | -0.006 | 0.004 | -1.608 | 0.108 |  |
| Hippocampus | -0.006 | 0.005 | -1.195 | 0.232 |  |
| Amygdala | 0.004 | 0.004 | 0.918 | 0.359 |  |
| Accumbens | 0.002 | 0.004 | 0.476 | 0.634 |  |
| Age | 0.001 | 0.002 | 0.233 | 0.816 |  |
| Sex | 0.005 | 0.004 | 1.450 | 0.147 |  |
| Deprivation/median family income | -0.708 | 0.008 | -90.941 | 0.000 |  |
| Stimulation/crime | 0.059 | 0.019 | 3.061 | 0.002 |  |
| Stimulation/population density | 0.111 | 0.008 | 13.344 | 0.000 |  |
| **Stimulation/crime** | β | SE | t-value | p-value |  |
| Intercept | 0.012 | 0.167 | 0.069 | 0.945 |  |
| Thalamus | 0.000 | 0.003 | 0.089 | 0.929 |  |
| Caudate | -0.003 | 0.003 | -1.081 | 0.280 |  |
| Putamen | -0.002 | 0.003 | -0.782 | 0.434 |  |
| Pallidum | 0.002 | 0.003 | 0.648 | 0.517 |  |
| Hippocampus | -0.001 | 0.003 | -0.357 | 0.721 |  |
| Amygdala | -0.003 | 0.003 | -0.910 | 0.363 |  |
| Accumbens | -0.001 | 0.003 | -0.231 | 0.817 |  |
| Deprivation/median family income | 0.018 | 0.004 | 4.070 | 0.000 |  |
| Discrepancy/income inequality | 0.023 | 0.004 | 5.420 | 0.000 |  |
| Stimulation/population density | 0.085 | 0.004 | 23.447 | 0.000 |  |
| Age | 0.000 | 0.002 | 0.086 | 0.932 |  |
| Sex | 0.003 | 0.002 | 1.227 | 0.220 |  |
| **Stimulation/population density** | β | SE | t-value | p-value |  |
| Intercept | 0.287 | 0.287 | 0.998 | 0.318 |  |
| Thalamus | 0.001 | 0.005 | 0.172 | 0.864 |  |
| Caudate | -0.001 | 0.004 | -0.123 | 0.902 |  |
| Putamen | 0.005 | 0.004 | 1.138 | 0.255 |  |
| Pallidum | 0.006 | 0.004 | 1.512 | 0.131 |  |
| Hippocampus | -0.011 | 0.005 | -2.276 | 0.023 |  |
| Amygdala | 0.003 | 0.005 | 0.542 | 0.588 |  |
| Accumbens | -0.008 | 0.004 | -1.955 | 0.051 |  |
| Deprivation/median family income | -0.107 | 0.012 | -9.001 | 0.000 |  |
| Discrepancy/income inequality | 0.133 | 0.011 | 11.991 | 0.000 |  |
| Stimulation/crime | 0.542 | 0.023 | 23.935 | 0.000 |  |
| Age | 0.001 | 0.003 | 0.557 | 0.578 |  |
| Sex | -0.002 | 0.004 | -0.586 | 0.558 |  |

**Supplementary figure 1**

*Flowchart indicating exclusion for analyses with baseline data, as well as for longitudinal analyses. MRI=magnetic resonance imaging, QC=quality control, FS=FreeSurfer, CBCL=child behavior checklist, PPS=prodromal questionnaire-brief child version, TP=timepoint. The ABCD Data Analysis and Informatics Center (DAIC) and the ABCD Imaging Acquisition Workgroup developed the imaging protocol. Scanning took place in 1-2 session, including 3D T1 and 3D T2-weighted images. Data collection occurred at 21 sites using 3 tesla scanners from three different vendors: Siemens, Phillips, and General Electric. Scanning models included Siemens Prisma, Siemens Prisma Fit, Phillips Achieva dStream, Phillips Ingenia, and General Electric Discovery MR750. Repetition time was 2400 to 2500 ms, echo time was 2-2.9 ms, field of view 256 x 240 x 256, FOV phase 93.75% to 100%, matrix 256 x 256, 176 to 225 slices, inversion delay 1060 ms, flip angle of 8°, voxel resolution of 1x1x1 mm, total acquisition ranging from 5 minutes 38 seconds to 7 minutes 12 seconds. DAIC performed processing steps in the Multi-Modal Processing Stream, a software package developed by the Center for Multimodal Imaging and Genetic at the University of California, San Diego. The pipeline includes: (1) preprocessing, which corrects for gradient nonlinearity distortions, intensity scaling and inhomogeneity correction, registration to an averaged reference brain in standard space, and manual quality control). (2) Brain segmentation, including cortical surface reconstruction and subcortical segmentation based on automatic, atlas-based, segmentation procedures in FreeSurfer v5.3 (Hagler Jr et al., 2019) (3) Calculation of morphometric measures using the Desikan-Killiany Atlas for cortical thickness and surface area, and the Aseg atlas for subcortical volumes (Fischl et al., 2002) (4) Post-processing quality control, which entailed manual review by trained technicians for motion, intensity inhomogeneity, white matter underestimation, pial overestimation, and magnetic susceptibility artifact (see (Hagler Jr et al., 2019) for further detail).*

**
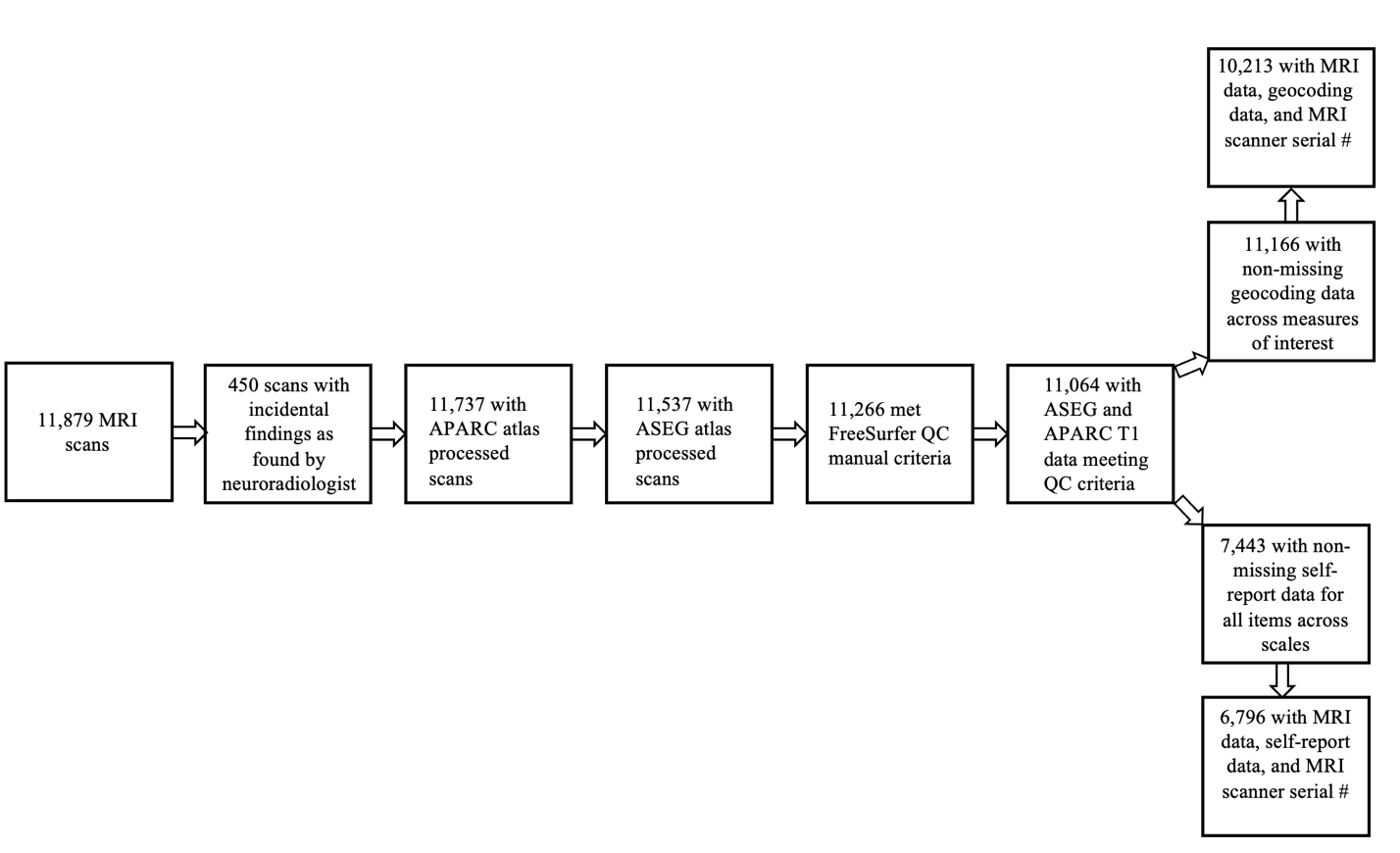
**

**Supplementary figure 2**

Quartile cutoffs and sample descriptions.

**
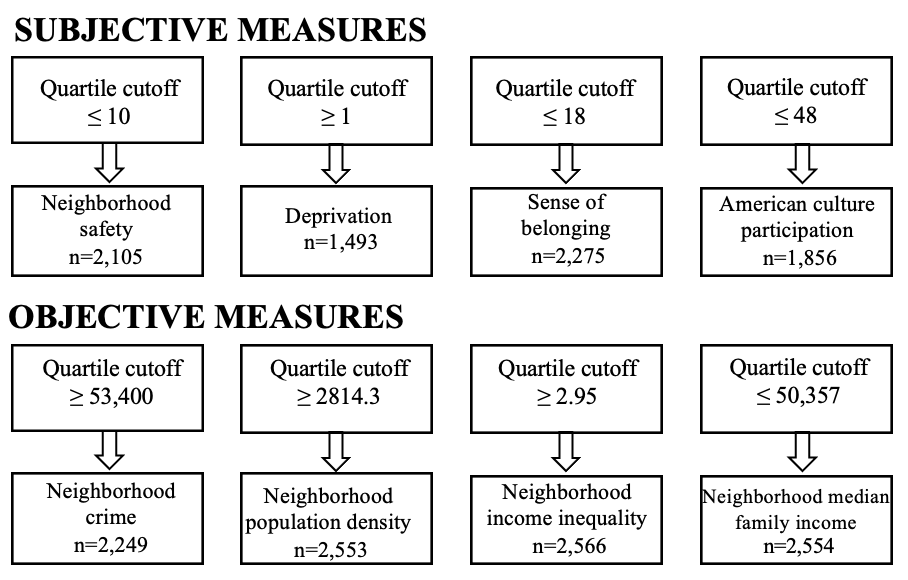
**

**Supplementary figure 3**

*Stress severity (as measured by the ABCD youth life events scale) and SDD domains (Grant, Compas, Thurm, McMahon, & Gipson, 2004; Tiet et al., 2001).*


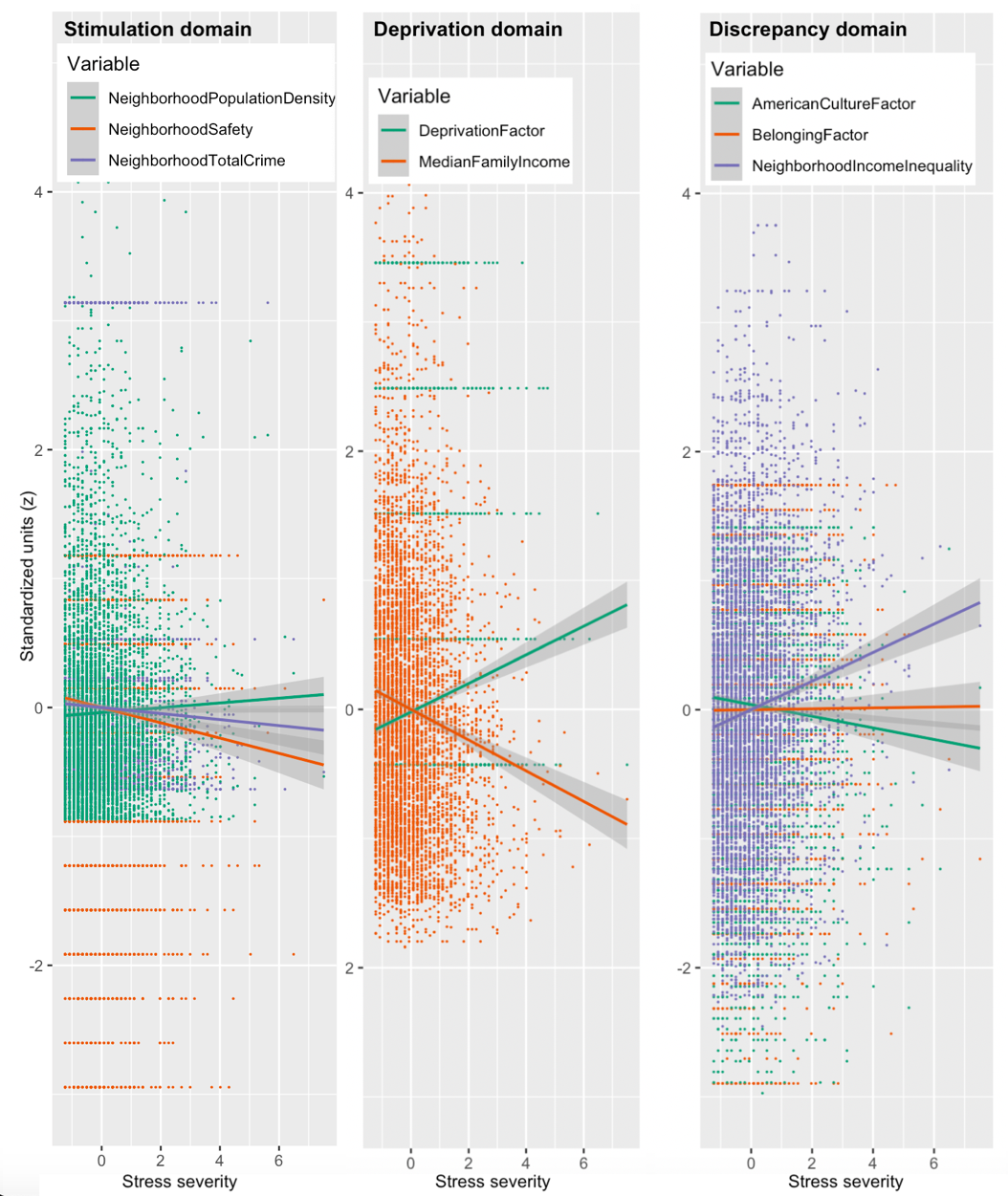
.
